# A Strategy for Simultaneous Isolation of Less Polar Ginsenosides, Including a Pair of New 20-Methoxyl Isomers, from Flower Buds of *Panax ginseng*

**DOI:** 10.3390/molecules22030442

**Published:** 2017-03-10

**Authors:** Sha-Sha Li, Ke-Ke Li, Fei Xu, Li Tao, Li Yang, Shu-Xiao Chen, Xiao-Jie Gong

**Affiliations:** 1 College of Medical, Dalian University, Dalian 116622, China; A956903967@163.com (S.-S.L.); xufei910324@163.com (F.X.); taoli020@126.com (L.T.); 2 College of Environmental and Chemical Engineering, Dalian University, Dalian 116622, China; chunmilier@126.com (L.Y.); 18340862946@163.com (S.-X.C.)

**Keywords:** 20(*S*)-methoxyl-ginsenoside Rg_3_, 20(*R*)-methoxyl-ginsenoside Rg_3_, isomer, quantification, flower buds of *Panax ginseng*

## Abstract

The present study was designed to simultaneously isolate the less polar ginsenosides from the flower buds of *Panax ginseng* (FBPG). Five ginsenosides, including a pair of new 20-methoxyl isomers, were extracted from FBPG and purified through a five-step integrated strategy, by combining ultrasonic extraction, Diaion Hp-20 macroporous resin column enrichment, solid phase extraction (SPE), reversed-phase high-performance liquid chromatography (RP-HPLC) analysis and preparation, and nuclear magnetic resonance (NMR) analysis. The quantification of the five ginsenosides was also discussed by a developed method with validations within acceptable limits. Ginsenoside Rg_5_ showed content of about 1% in FBPG. The results indicated that FBPG might have many different ginsenosides with diverse chemical structures, and the less polar ginsenosides were also important to the quality control and standardization of FBPG.

## 1. Introduction

*Panax ginseng* (PG) C. A. Meyer, well-known as “renshen” in Chinese, is one of the most famous medicinal herbs and used widely as an herbal remedy and healthy food for thousands of years [1]. Ginsenosides are considered as the major active component of PG with multiple pharmacological activities [2,3,4]. Up to now, more than 100 naturally-occurring ginsenosides have been isolated from roots, leaves/stems, fruits, and/or flower buds of PG [5,6,7,8]. In traditional Chinese medicine, PG root is the most commonly used part [9,10,11,12]. Due to its significant tonic and restorative effects, more attention was paid to PG. In addition to PG root, such as leaves and stems, flower buds and fruits were also studied for their medicinal values [13,14,15]. Recently, the ginsenosides in the flower buds of *Panax ginseng* (FBPG) gradually attracted people’s attention, and it was considered as a valuable functional food with medicinal potential. People commonly use it as tea for anti-fatigue and for enhancing immunity [16].

Due to the higher content of total ginsenosides in FBPG than the other parts of PG [17,18], to find new active compounds, further isolation and identification studies of constituents in FBPG were carried out. Some new and minor ginsenosides, such as ginsenoside Rd, floralginsenoside Ka, floralginsenoside Ta, ginsenoside Rk_3_, and ginsenoside Rs_4_, have been identified with extensive bioactivities, including gastroprotective effect [19], radical scavenging [20], and anti-tumor [14] and anti-inflammatory activity [21]. In order to extend the utility value of FBPG, it is necessary to systematically uncover its chemical basis. PG contains many complex and structurally similar ginsenosides. Thus, multidisciplinary methods are required to analyze and identify its genuine structures. With the development of liquid chromatography coupled to mass spectrometry (LC-MS) [22,23], and assisted by the proper sample pre-preparation methods, it has become relatively simple and efficient to study ginsenosides.

**Figure 1:**
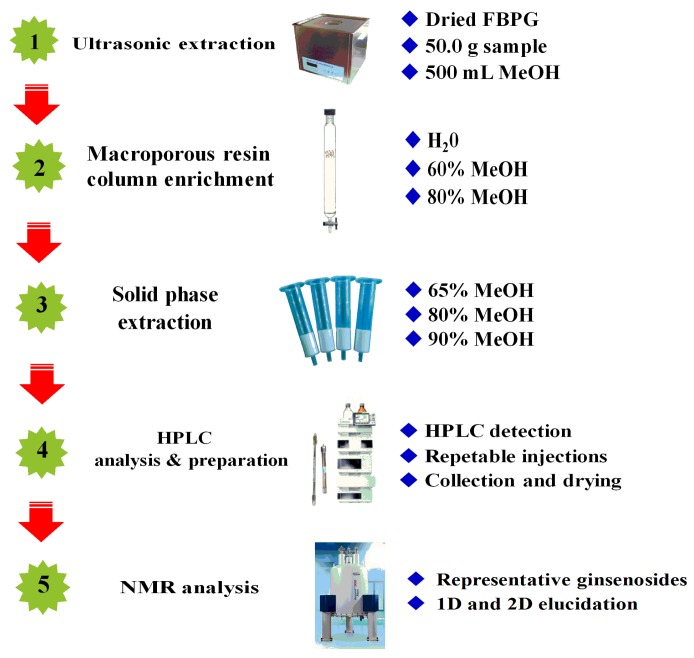
A flowchart of our integrated strategy for isolation and purification of novel ginsenosides from the flower buds of *Panax ginseng* (FBPG). HPLC, high-performance liquid chromatography.

**Figure 2:**
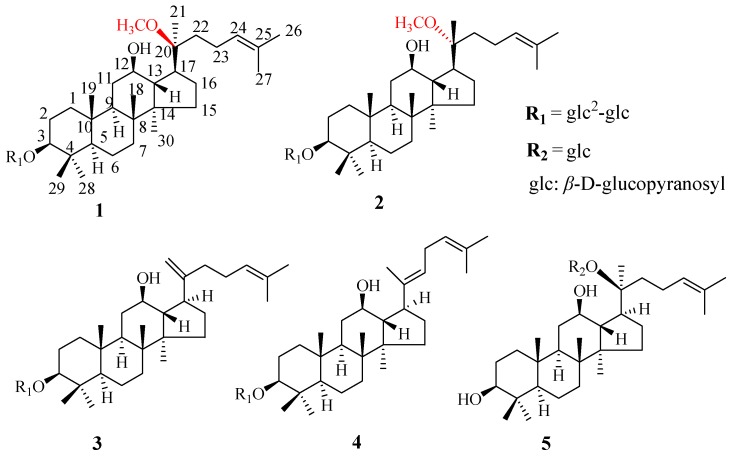
Chemical structures of ginsenosides **1**–**5**.

Based on our previous work on the isolation and determination of the isomeric compounds ginsenoside F_5_ and F_3_ from FBPG [24], in this study, a five-step integrated strategy, by combining ultrasonic extraction, Diaion Hp-20 macroporous resin column enrichment, solid phase extraction (SPE), reversed-phase high-performance liquid chromatography (RP-HPLC) analysis and preparation, and NMR analysis, is presented (Figure 1). We obtained five less polar ginsenosides, including a pair of new isomeric ginsenosides (Figure 2). Moreover, the quantification of the five ginsenosides in FBPG was also involved. This research will provide a novel scientific method for comprehensive studies of various ginsenosides in FBPG and make full use of ginseng resources.

## 2. Results and Discussion

### 2.1. Purification of the Five Less Polar Ginsenosides from the Flower Buds of Panax Ginseng

#### 2.1.1. Preparation of Crude Ginsenoside Extracts

The most appropriate and simple method for extracting less polar ginsenosides from the ginseng plant is ultrasound-assisted extraction [25,26]. Methanol was used as the solvent, and it was tested under the following conditions: a sample of 50.0 g, a solvent to solid ratio of 10 mL/g, a temperature of 30 °C, ultrasound power of 1000 W, an extraction time of 30 min, a particle size of 80–100 mesh, and it was extracted three times. The combined extract was concentrated under vacuum yielding 8.2 g (16.4%) of crude extract powder. To obtain the high yield of less polar ginsenoside from FBPG extracts, Diaion HP-20 macroporous resin was used to remove the polar ginsenosides by the elution with H_2_O and 60% aqueous methanol, followed by 80% aqueous methanol. The last fraction was rich in less polar ginsenosides (Figure S1), which were used to further isolate and purify specific ginsenosides.

#### 2.1.2. Optimization of Solid Phase Extraction Conditions

In order to simplify the procedure of the semi-preparative liquid chromatography separation, it is important to obtain a relatively uncomplicated sample. It might achieve good results of isolation under fewer steps. Based on the HPLC chromatogram (Figure S1), a simple and efficient method was established for rapid purification of the crude ginsenoside extracts by SPE.

**Figure 3:**
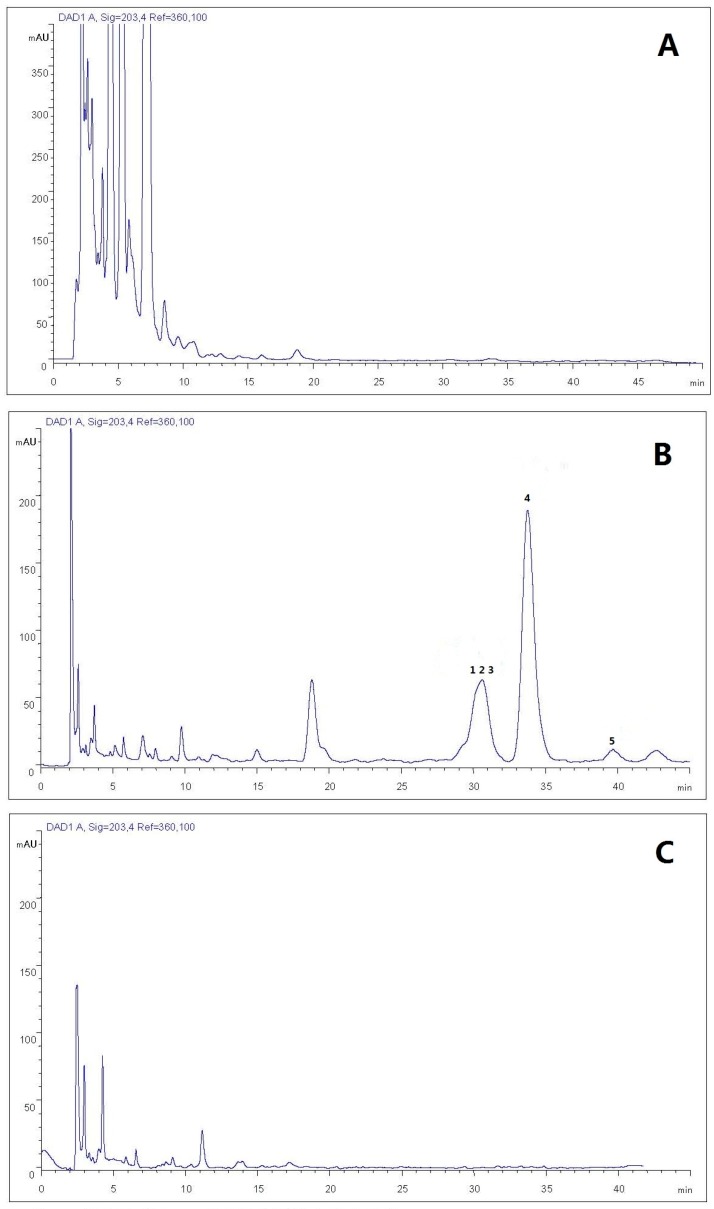
The chromatograms of ginsenoside fractions obtained from solid phase extraction (SPE) with different eluting solvents (methanol/water = 80:20). (**A**) The eluted fraction of 65% aqueous methanol; (**B**) the eluted fraction of 80% aqueous methanol; and (**C**) the eluted fraction of 90% aqueous methanol. Peaks: 1: 20(*S*)-methoxyl-ginsenoside Rg_3_; 2: 20(*R*)-methoxyl-ginsenoside Rg_3_; 3: ginsenoside Rk_1_; 4: ginsenoside Rg_5_; 5: ginsenoside M1.

In this section, three different strategies were used to test the efficiency of the elution. Crude ginsenoside powder (102.5 mg) was dissolved in 2.0 mL methanol and was subjected to C18 Sep-Pak^®^ cartridge column chromatography with a stepwise gradient methanol–water. The first step was methanol–water (40:60, 65:35, 80:20, 90:10 *v*/*v*), the second step was methanol–water (68:32, 80:20, 90:10 *v*/*v*), and the third step was methanol–water (65:35, 80:20, 90:10 *v*/*v*) (6.0 mL solvent per step, performed three times). By comparing the peaks in terms of the five less polar ginsenosides in the chromatogram, the majority of five less polar ginsenosides absorbed by the SPE column was eluted by methanol–water (80:20) solution (Figure 3B) after it was firstly eluted by methanol–water (65:35) solution (Figure 3A), where complete desorption was conducted with an elution volume of 18 mL. We could prove that a good desorption was achieved by methanol–water (65:35, 80:20 *v*/*v*) since there was no target compound in the 90% aqueous methanol fraction (Figure 3C). However, when the methanol–water (68:32, 80:20 *v*/*v*) was used as the elution, the target compounds were also eluted together with the polar ginsenosides by methanol-water (68:32 *v*/*v*) (Figure S2). Therefore, a stepwise gradient methanol–water (65:35, 80:20, 90:10 *v*/*v*) was selected as the eluent in the SPE processing section for preparation of samples for the semi-preparative liquid chromatography separation.

#### 2.1.3. Establishment of HPLC Isolation Conditions

The chromatogram in Figure 3B shows the isolated peaks after the SPE treatment, but a satisfactory separation was not obtained with a mobile phase composed of methanol-water, especially for compounds **1**–**3**. Usually the ginsenosides could achieve a complete separation effect with acetonitrile-water as the mobile phase [27,28]. Using acetonitrile-water, we discuss the parameters of volume ratios, flow rate, and column temperature here.

**Figure 4:**
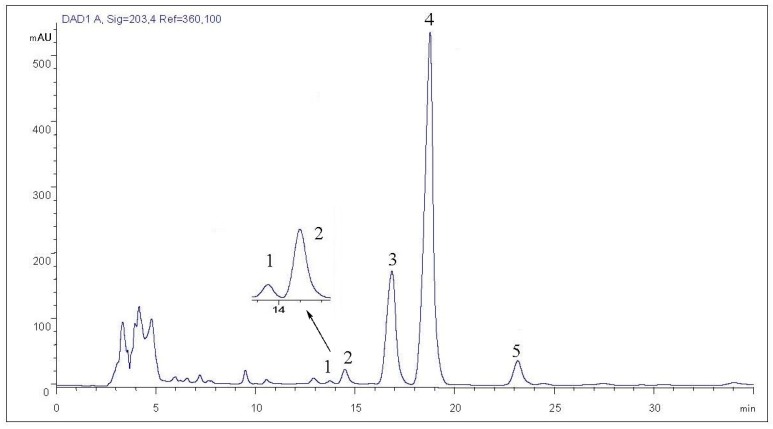
The chromatogram of ginsenoside fractions obtained from SPE with 80% methanol (acetonitrile/water = 53:47, flow rate 0.6 mL/min, column temperature 15 °C).

The best separation was obtained with the system of acetonitrile/water (53:47 *v*/*v*), as shown in Figure 4. With the system of acetonitrile/water (50:47 *v*/*v*), the two peaks, ginsenosides Rg_5_ and M1, overlapped each other completely (Figure S3).

Different flow rate could also affect the separation effect of ginsenoside compounds. With the mobile phase of acetonitrile/water (53:47 *v*/*v*), the flow rate of 0.5 mL/min (Figure S4) was satisfactory, and the analytical time was less than 30 minutes. While the flow rate of 0.6 mL/min led to partial overlapping of the two new isomeric ginsenosides, ginsenoside M1 was also affected by the adjacent compounds (Figure 4).

As for the influence of column temperature, the lower column temperature should be adjusted to meet the separation needs of these target compounds. As shown in Figure S5, the column temperature of 25 °C resulted in a poor separation effect, and when it was set at 15 °C (Figure S4), the five ginsenosides were all separated in good condition.

According to the analytical method, the separation process was scaled up to preparative-scale chromatography to obtain a significant quantity of the five ginsenosides. Using the semi-preparative HPLC Daisogel C18 column (5 μm, 30 mm internal diameter (i.d.) × 250 mm) column, we obtained five pure specific ginsenosides with a mobile phase of acetonitrile/water (53:47 *v*/*v*) at a flow rate of 5.0 mL/min within 30 min. The typical chromatogram of the semi-HPLC method is illustrated in Figure S6.

### 2.2. Identification of the Isolated Ginsenosides

Compound **1** was obtained as a colorless amorphous powder. The molecular formula was deduced to be C_43_H_74_O_13_ on the basis of the protonated molecular ion peak in positive high resolution electrospray ionization mass spectrometry (HR-ESI-MS) at *m*/*z* 799.5195 ([M + H]^+^, calcd. 799.5208). Its IR spectrum displayed strong absorption bands at 1636 cm*^−^*^1^, indicating an olefinic functional group, and at 3397 and 1080 cm*^−^*^1^ suggestive of hydroxyl and ether functions. The ^1^H-NMR spectrum of **1** displayed signals due to eight tertiary methyls, an olefin, and two anomeric protons (Table 1). The ^1^H- and ^13^C-NMR data of **1** were very similar to those of 20(*S*)-ginsenoside Rg_3_ [29], except for signals indicating the presence of an extra methoxyl group (δ H 3.27; δ C 48.8), suggesting that **1** was a methyl ether of 20(*S*)-ginsenoside Rg_3_. The methoxyl group could be located at C-20 based on the downfield shift of C-20 (δ 79.9) and the highfield shifts of C-17 (δ 50.0), C-21 (δ 21.2) and C-22 (δ 35.1), which appeared at δ 73.0 (C-20), δ 54.8 (C-17), δ 27.1 (C-21), and δ 35.9 (C-22) in 20(*S*)-ginsenoside Rg_3_ [29], respectively. This was further supported by the long-range correlation between the methoxyl signal at δ 3.27 (OCH_3_) and the carbon signal at δ 79.9 (C-20) in the HMBC spectrum. The detailed NMR data was assigned by the heteronuclear singular quantum correlation (HSQC) and heteronuclear multiple bond correlation (HMBC) spectrum (Table 1).

Compound **2** was also obtained as a colorless amorphous powder. The molecular formula was deduced to be C_43_H_74_O_13_ on the basis of the protonated molecular ion peak in positive HR-ESI-MS at *m*/*z* 799.5194 ([M + H]^+^, calcd. 799.5208). It had the same molecular weight as compound **1**. Its IR spectrum showed identical features as that of **1**. The ^13^C-NMR spectrum gave 43 carbon signals, of which 12 were assigned to the sugar moiety and 31 to a triterpene aglycone moiety. The NMR data assignments were elucidated by the HSQC and HMBC spectrum. By comparing NMR data of **2** with those of **1**, few differences were found due to the ^13^C-NMR data of C-17, C-20, C-21, and C-22 (Table 1). Compounds **1** and **2** were a pair of isomers with different stereochemistry configuration at C-20.

**Figure 5:**
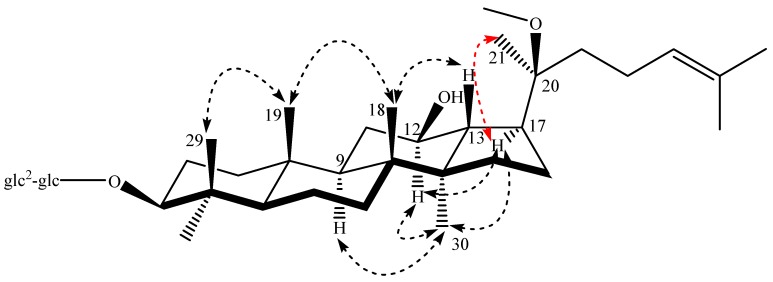
Key NOE correlations for new compound **1**.

The determination of the configuration at C-20 was based on the rotating frame overhauser effect spectroscopy (ROESY) experiment. In compound **1**, the coupling constant between H-13 and H-17 (*J* = 11.0 Hz) and the key nuclear overhauser effect (NOE) correlations from H-13 (δ_H_ 1.86) to H-18 (δ_H_ 0.95) and from H-17 (δ_H_ 2.42) to H_3_-21 (δ_H_ 1.19) and H_3_-30 (δ_H_ 0.96) (Figure 5) indicated the assignments of the α-oriented H-17 and of the C-20(*S*) configuration [30]. However, in compound **2**, we did not observe the key NOE correlation from H-17 (δ_H_ 2.35) to H_3_-21 (δ_H_ 1.17), and the other correlations were the same as compound **1**, the coupling constant between H-13 and H-17 showed 10.5 Hz, and all of these supported the α-oriented H-17 and C-20(*R*) configuration [31]. Thus, the structure of the new compound **1** was unambiguously formulated as 3β,12β-dihydroxy-20(*S*)-methoxyl-dammar-24-ene 3-*O*-β-d-glucopyranosyl-(1→2)-β-d-glucopyranoside and given the trivial name 20(*S*)-methoxyl-ginsenoside Rg_3_. The structure of new compound **2** was identified as 3β,12β-dihydroxy-20(*R*)-methoxyl-dammar-24-ene 3-*O*-β-d-glucopyranosyl-(1→2)-β-d-glucopyranoside, and named 20(*R*)-methoxyl-ginsenoside Rg_3_.

The three known compounds, ginsenoside Rk_1_ (**3**), ginsenoside Rg_5_ (**4**), and ginsenoside M1 (**5**), were readily identified by comparison of their data with the literature [32,33]. To the best of our knowledge, they were the first scientific report from FBPG.

^13^C-NMR data played a very important role in the determination of C-20 ginsenoside isomers. We could make a distinction between 20(*S*) and 20(*R*) by the ^13^C-NMR data at C-17, C-20, C-21, and C-22. Sometimes C-20 did not show the difference [29,34], but the data at C-17, C-21, and C-22 are always different and could be used to distinguish 20(*S*) from 20(*R*). Its chemical shift differences are shown in Table 2.

We could also distinguish 20(*S*) from the 20(*R*) ginseoside isomer by the retention time in RP-HPLC. Usually, the retention time of the 20(*S*) isomer was shorter than the 20(*R*) isomer [34,35]. In this paper, our study validated the correct retention property of the isomers on the C18 column.

Optical rotation for the two new isomers was analyzed in dimethyl sulfoxide (DMSO) and/or MeOH due to differences in solubility. Like the positive data of 20(*S*)-ginsenoside Rg_3_ previously reported [34], we obtained a positive value in DMSO and MeOH for 20(*S*)-methoxyl-ginsenoside Rg_3_, and there was also a negative value reported for 20(*S*)-ginsenoside Rg_3_ in DMSO [36]. However, according to the synthesis method of 20(*S*)-ginsenoside Rg_3_ with the positive value in MeOH [37], we prove that, at least, 20(*S*)-methoxyl-ginsenoside Rg_3_ shows positive optical rotation in MeOH. Since 20(*R*)-methoxyl-ginsenoside Rg_3_ was not soluble in MeOH, just like 20(*R*)-ginsenoside Rg_3_, we could not observe its optical rotation in MeOH.

It was reported that similar C-20 methyl ether compound has been isolated from ginseng genus plants, such as *Panax vietnamensis* [38]. Thus, our findings further proved that this kind of ginsenoside was native to the plant. Together, they made up a wide variety of chemical structures of ginsenosides. Furthermore, the variations observed in the C-17 side-chain of ginsenosides were usually considered as artifacts, which were produced through the processed ginseng [8], but some ginsenosides have been also isolated from the natural plant, such as the stems and leaves [32]. This might indicate that the aerial part of PG, including flower buds, and stems and leaves, had more abundant compounds of structural changes than roots due to the long time in sunlight and high temperature. Thus, it is important to analyze these less polar ginsenosides in FBPG for quality control.

### 2.3. Method Validation of Quantification

All calibration curves were constructed by plotting the peak areas versus the concentration and showed good linear regression (*R*^2^ > 0.9994) within the test ranges. The limits of detection (LODs) (signal-to-noise ratio, S/N = 3) and the limits of quantification (LOQs) (S/N = 10) for the five ginsenosides were less than 0.092 and 0.275 μg, respectively (Table 3). The overall intra- and inter-day variations were within 0.35%–1.51% and 0.41%–1.54% for the five analytes (Table 4). As shown in Table 5, the developed analytical method had an excellent accuracy with an overall recovery from 97.20% to 99.71% (*n* = 3) for the analytes. All of the above indicated that this high-performance liquid chromatography diode array detector (HPLC-DAD) method was precise, accurate, and sensitive enough for the simultaneous quantitative evaluation of the five ginsenosides in FBPG.

### 2.4. Quantitative Analysis of the Five Less Polar Ginsenosides in FBPG

Ten grams (dry weight) of FBPG sample was extracted in triplicate by ultrasound-assisted extraction with methanol (each 100 mL, *w*/*v* = 1:10). Then the extracts were treated as described in Section 2.1 by a Diaion HP-20 macroporous resin column and C18 cartridges column to prepare the sample for HPLC analysis. The residue from SPE was concentrated and dissolved in methanol with a 10 mL volumetric flask, then filtered through a 0.45 μm syringe filter. This was conducted with three parallel tests to obtain three solutions, and each solution was analyzed three times. The content of the five less polar ginsenosides in FBPG was shown in Table 6.

## 3. Materials and Methods

### 3.1. General

Ultrasound-assisted extraction was carried out in a SB-5200DTD ultrasonic device (Ningbo Xinzhi Biotechnology Co., Ltd., Ningbo, China). Open column chromatography was carried out using a Diaion HP-20 (Mitsubishi Chemical Corp., Fuji, Tokyo, Japan) as the stationary phase. C18 Sep-Pak^®^ cartridge (12 cc/2 g) columns were obtained from Waters (Milford, MA, USA). Double-distilled water was used in all experiments and samples for HPLC were filtered through a 0.45 μm membrane before injection. HPLC was carried out on an Agilent Series 1260 (Agilent Technologies, Palo Alto, CA, USA) liquid chromatography system, equipped with a vacuum degasser, a quaternary pump, a column oven, a 7125i Reodyne Model manual injector with a 20 μL loop, and a diode array detector. The semi-preparative HPLC was performed on a CXTH HPLC system (Chuang Xin Tong Heng Sci. Technol. Co. Ltd., Beijing, China) equipped with two P3050 pumps, a UV-3000 UV-VIS detector (Chuang Xin Tong Heng Sci. Technol. Co. Ltd., Beijing, China), and a 7125i Reodyne Model manual injector with a 5 mL loop. The IR spectra were recorded as KBr pellets on a Jasco 302-A spectrometer (Jasco, Tokyo, Japan). Optical rotation was recorded on a Jasco P-2000 polarimeter. NMR spectra were measured on a Bruker DRX500 spectrometer (Bruker S.A., Wissembourg, France) with tetramethyl silicane (TMS) as internal standard. HR-ESI-MS were measured on a 6550 Agilent iFunnel Q-TOF LC/MS (Agilent Technologies). The melting point was recorded on an X-4 microscopic melting point meter (uncorrected) (Maisiqi High-tech Co. Ltd., Beijing, China). All of the reagents were of analytical grade bought from Sinopharm Chemical Reagent Co., Ltd. (Shanghai, China).

### 3.2. Plant

The FBPG were collected from Tonghua County (Jilin, China) in 2014 and identified by one of the authors, Professor Xiao-Jie Gong.

### 3.3. Sample Preparation

An ultrasonic extraction method was employed. In brief, the air-dried 1 kg particle size of 80–100 mesh powder of FBPG was ultrasonically extracted in 10 L of methanol on a water bath (1000 W) at 30 °C for 30 min, repeating the extraction operation two times. After removing the solvents under vacuum, the extract was dissolved in a small amount of water and then chromatographed on a Diaion HP-20 column eluted with H_2_O, 60% aqueous methanol, and 80% aqueous methanol. The last fraction with enriched less polar ginsenosides was collected for further processing by SPE. The elution was carried out with increased concentrations of methanol (65%, 80%, and 90%, 6 mL each, for three times) successively. The eluted fraction of 80% aqueous methanol was rich in our targeted ginsenosides. It was ready for the isolation of specific compounds and quantification.

### 3.4. HPLC Analysis and Preparation

The analytical HPLC conditions was selected as: Agilent Zorbax Eclipse XDB C-18 (5 μm; 4.6 mm i.d. × 250 mm) column, mobile phase of acetonitrile/water (53:47 *v*/*v*), flow rate of 0.5 mL/min, column temperature of 15 °C, detected at 203 nm. The semi-preparative HPLC method used to purify the five ginsenosides was: Daisogel C-18 column (5 μm, 20 mm i.d. × 250 mm, Microwants, Suzhou, China), mobile phase of acetonitrile/water (53:47 *v*/*v*), flow rate of 5.0 mL/min, column temperature of 15 °C, detected at 203 nm. Finally, it gave **1** (1.1 mg), **2** (25.6 mg), **3** (110.5 mg), **4** (865.2 mg), and **5** (64.8 mg) from 1 kg FBPG after repeated injection.

### 3.5. Structural Characterization of New Ginsenosides

*20(S)-methoxyl-ginsenoside Rg_3_:* colorless amorphous powder. m.p.: 275–278 °C. ${\left[\alpha \right]}_{D}^{20}$ +0.29° (*c* 1.0 DMSO) and ${\left[\alpha \right]}_{D}^{20}$ +10.62° (*c* 1.0 MeOH). HR-ESI-MS (*m*/*z*): 799.5195 [M + H]^+^ (calcd. C_43_H_75_O_13_ 799.5208), 821.5009 [M + Na]^+^ (calcd. C_43_H_74_O_13_Na 821.5027). IR (KBr) 3397, 2938, 1636, 1383, 1080, and 1033 cm^−1^; ^1^H- and ^13^C-NMR (C_5_D_5_N) data, see Table 1.

*20(R)-methoxyl-ginsenoside Rg_3_*: colorless amorphous powder. m.p.: 211–214 °C. ${\left[\alpha \right]}_{D}^{20}$ −0.88° (*c* 1.0 DMSO). HR-ESI-MS (*m*/*z*): 799.5194 [M + H]^+^ (calcd. C_43_H_75_O_13_ 799.5208), 821.5012 [M + Na]^+^ (calcd. C_43_H_74_O_13_Na 821.5027). IR (KBr) 3395, 2942, 1646, 1385, 1079, and 1038 cm^−1^; ^1^H- and ^13^C-NMR (C_5_D_5_N) data, see Table 1.

### 3.6. Acid Hydrolysis

A solution of compounds **1** and **2** (1 mg each) in 1 M HCl (1.0 mL) was stirred for 4 h at 80 °C to release the sugar moiety by a previously reported method [5]. After the extraction with EtOAc, the concentrated aqueous layer was analyzed by the following HPLC conditions: Kaseisorb LC NH2-60-5 column (5 μm; 4.6 mm i.d. × 250 mm, Tokyo Kasei Co. Ltd., Tokyo, Japan); optical rotation detection; and mobile phase of acetonitrile/water (75:25 *v*/*v*). Peaks of the hydrolysate were detected by comparison with retention time of authentic samples of d-glucose (12.3 min).

### 3.7. Validation

Six concentrations of compounds **1**–**5** solutions were injected in triplicate, and then the calibration curves were constructed by plotting the peak areas against the concentration of each analyte. The diluted solutions of the analytes were further diluted with methanol to give a series of concentrations for determining the LODs and LOQs. The LOD and LOQ for individual ginsenosides in FBPG under present chromatographic conditions were determined on the basis of the responses at a S/N of 3 and 10, respectively. The mixed standard samples were chosen to determine intra- and inter-day precision of the method and analyzed as described in Section 2.1.3. The intra-day precision was performed in triplicate on a single day. The inter-day precision was carried out on five different days. The concentrations were calculated from the corresponding calibration curve. Variations were expressed by the RSD. Accurate amounts of standard ginsenosides were added to an accurately-weighed portion of FBPG, and then extracted and analyzed as described above.

## 4. Conclusions

An efficient simultaneous purification method combining SPE chromatography with semi-preparative HPLC was established to isolate five less polar ginsenosides from FBPG in the present study. The purified ginsenosides included a pair of new 20-methoxyl isomers and three known compounds which were identified for the first time from FBPG. The new compounds were elucidated by NMR and MS analysis. Furthermore, a rapid and efficient RP-HPLC method was established for systematic quantitative analysis of the five less polar ginsenosides in FBPG. Ginsenoside Rg_5_ was recognized as the relatively most abundant (approximate 1%) in FBPG, which could also be selected as a marker compound for quality control, except for the polar ginsenosides, such as ginsenoside Re and ginsenoside Rd. The developed RP-HPLC method demonstrated an effective analytical means for less polar ginsenosides in FBPG. The results could provide a new material chemistry basis for the further complete application of FBPG in functional food with medicinal properties.

## Figures and Tables

**Table 1:** ^1^H- and ^13^C-NMR data for **1** and **2** (500 and 125 MHz, C_5_D_5_N, *J* in Hertz, and δ in ppm).

Position	1		2	
	δ_H_	δ_C_	δ_H_	δ_C_
1	0.87 (1H, m) 1.58 (1H, m)	39.2	0.78 (1H, m) 1.52 (1H, m)	39.2
2	1.24 (1H, m) 1.86 (1H, m)	26.3	1.29 (1H, m) 1.89 (1H, m)	26.3
3	3.32 (1H, dd, 12.0, 4.5)	89.0	3.32 (1H, dd, 11.5, 4.0)	89.0
4	-	39.7	-	39.7
5	0.73 (1H, br d, 12.0)	56.4	0.72 (1H, br d, 11.5)	56.4
6	1.32 (1H, m) 1.47 (1H, dd, 12.0, 4.2)	18.5	1.40 (1H, m) 1.52 (1H, dd, 12.0, 4.2)	18.5
7	1.22 (1H, m) 1.51 (1H, m)	35.2	1.24 (1H, m) 1.47 (1H, m)	35.2
8	-	40.0	-	40.1
9	1.41 (1H, m)	50.0	1.43 (1H, m)	50.2
10	-	37.0	-	37.0
11	1.38 (1H, m) 2.03 (1H, m)	31.2	1.45 (1H, m) 2.04 (1H, dd, 12.0, 7.0)	31.4
12	3.76 (1H, dt, 9.5, 5.5)	70.4	3.81 (1H, dt, 10.0, 3.0)	70.6
13	1.86 (1H, t, 11.0)	49.0	1.83 (1H, t, 10.5)	49.7
14	-	51.5	-	51.8
15	1.05 (1H, t, 11.0) 1.57 (1H, m)	30.9	1.05 (1H, t, 10.0) 1.54 (1H, m)	31.3
16	1.73 (1H, m) 2.23 (1H, m)	26.8	1.85 (1H, m) 2.22 (1H, m)	26.8
17	2.42 (1H, td, 9.0)	50.0	2.35 (1H, td, 7.5)	47.2
18	0.95 (3H, s)	15.9	1.00 (3H, s)	15.9
19	0.83 (3H, s)	16.3	0.86 (3H, s)	16.4
20	-	79.9	-	80.2
21	1.19 (3H, s)	21.2	1.17 (3H, s)	18.5
22	1.67 (1H, m) 2.04 (1H, m)	35.1	1.44 (1H, m) 1.66 (1H, m)	35.7
23	2.05 (1H, m) 2.30 (1H, m)	22.8	2.13 (1H, m) n.d.	21.7
24	5.25 (1H, t, 6.5)	125.5	5.23 (1H, t, 7.5)	125.0
25	-	131.3	-	131.4
26	1.67 (3H, s)	25.8	1.75 (3H, s)	25.8
27	1.64 (3H, s)	17.7	1.67 (3H, s)	17.7
28	1.32 (3H, s)	28.2	1.33 (3H, s)	28.2
29	1.13 (3H, s)	16.7	1.14 (3H, s)	16.6
30	0.96 (3H, s)	17.1	0.98 (3H, s)	17.4
20-OCH_3_	3.27 (3H, s)	48.8	3.20 (3H, s)	48.4
1′	4.95 (1H, d, 7.5)	105.1	4.96 (1H, d, 7.5)	105.2
2′	4.26 (1H, m)	83.5	4.25 (1H, m)	83.5
3′	4.28 (1H, m)	78.0	4.27 (1H, m)	78.0
4′	4.18 (1H, t, 10.0)	71.7	4.17 (1H, t, 9.0)	71.7
5′	3.96 (1H, m)	78.3	3.96 (1H, m)	78.3
6′	4.50 (1H, m) 4.58 (1H, br d, 11.0)	62.9	4.49 (1H, m) 4.58 (1H, br d, 11.5)	62.9
1″	5.40 (1H, d, 7.5)	106.1	5.41 (1H, d, 7.5)	106.1
2″	4.16 (1H, t, 10.0)	77.2	4.16 (1H, t, 9.0)	77.2
3″	4.33 (1H, m)	78.4	4.34 (1H, m)	78.4
4″	4.36 (1H, m)	71.8	4.36 (1H, m)	71.8
5″	3.94 (1H, m)	78.2	3.94 (1H, m)	78.1
6″	4.32 (1H, m) 4.50 (1H, m)	62.8	4.38 (1H, br d, 11.5) 4.50 (1H, m)	62.8

n.d.: not detected.

**Table 2:** Chemical shift differences of C-17, C-20, C-21, and C-22 between 20(*R*) and 20(*S*) isomer ginsenosides (in C_5_D_5_N).

Ginsenoside	C-17	C-20	C-21	C-22
**20(*R*)-methoxyl-ginsenoside Rg_3_**	**47.2**	**80.2**	**18.5**	**35.7**
**20(*S*)-methoxyl-ginsenoside Rg_3_**	**50.0**	**79.9**	**21.2**	**35.1**
**Δ_20(*R*)-20(*S*)_δ**	**−2.8**	**0.3**	**−2.7**	**0.6**
20(*R*)-ginsenoside Rg_3_ [27]	50.7	73.0	22.8	43.3
20(*S*)-ginsenoside Rg_3_	54.8	73.0	27.1	35.9
Δ_20(*R*)-20(*S*)_δ	−4.1	0	−4.3	7.4
20(*R*)-ginsenoside Rf_2_ [27]	51.8	73.4	22.9	43.6
20(*S*)-ginsenoside Rf_2_	54.7	72.5	27.2	36.4
Δ_20(*R*)-20(*S*)_δ	−2.9	0.9	−4.3	7.2
20(*R*)-ginsenoside Rh_2_ [27]	52.2	73.4	23.0	43.7
20(*S*)-ginsenoside Rh_2_	54.8	72.9	26.9	35.2
Δ_20(*R*)-20(*S*)_δ	−2.6	0.5	−3.9	8.5

**Table 3:** Calibration curves and LODs for the five less polar ginsenosides.

Compounds *	Calibration Curve	Correlation Coefficient (*R*^2^)	Test Range (μg/mL)	LOD (μg)	LOQ (μg)
**1**	*y* = 2359.3*x* + 65.67	0.9998	8–80	0.086	0.262
**2**	*y* = 2714.9*x* + 46.11	0.9998	100–1000	0.074	0.275
**3**	*y* = 6665.5*x* + 270.39	0.9998	400–1500	0.092	0.258
**4**	*y* = 3332.6*x* + 139.30	0.9999	1000–5000	0.069	0.232
**5**	*y* = 2640.6*x* + 140.72	0.9994	100–1000	0.088	0.263

* Compounds **1**–**5** were the same as in the HPLC chromatogram. LOD, limit of detection; LOQ, limit of quantification.

**Table 4:** Precision of the HPLC-DAD method for the five less polar ginsenosides.

Compounds	Precision			
	Intra-Day (*n* = 3)		Inter-Day (*n* = 5)	
	Content (μg/mL)	RSD (%)	Content (μg/mL)	RSD (%)
**1**	80.34 ± 1.02	1.51	80.58 ± 1.24	1.54
**2**	200.26 ± 1.52	0.76	200.47 ± 1.82	0.91
**3**	501.72 ± 2.55	0.51	500.29 ± 2.78	0.56
**4**	1401.89 ± 4.93	0.35	1401.25 ± 5.78	0.41
**5**	203.57 ± 1.58	0.78	202.15 ± 2.51	1.24

HPLC-DAD; high-performance liquid chromatography diode array detector; RSD, relative standard deviation.

**Table 5:** Accuracy of HPLC method for the determination of the five less polar ginsenosides.

Compounds	Original (μg)	Spiked (μg)	Found (μg)	Recovery (%)	RSD (%)
**1**	5.70	35.00	40.58 ± 0.41	97.89	1.00
**2**	106.92	150.00	256.26 ± 2.08	99.38	0.81
**3**	512.03	500.00	1010.06 ± 5.23	99.61	0.52
**4**	3123.73	400.00	3521.27 ± 5.65	99.92	0.16
**5**	240.24	200.00	439.16 ± 5.36	99.54	1.22

**Table 6:** The content of the five less polar ginsenosides in FBPG.

Compounds	Content (μg/g FBPG) (*n* = 9) (mean ± SD)
**1**	1.77 ± 0.93
**2**	33.26 ± 7.46
**3**	159.31 ± 17.12
**4**	971.28 ± 28.05
**5**	74.65 ± 8.42

## Supplementary Materials

Supplementary materials are available online. Figure S1. The chromatogram of crude ginsenoside extracts of FBPG after treatment with a Diaion HP-20 macroporous resin column (methanol/water = 80:20); Figure S2. The chromatogram of the ginsenoside fraction obtained from SPE with 68% methanol (methanol/water = 80:20); Figure S3. The chromatogram of the ginsenoside fraction obtained from SPE with 80% methanol (acetonitrile/water = 50:47, flow rate: 0.6 mL/min, column temperature: 15°C); Figure S4. The chromatogram of the ginsenoside fraction obtained from SPE with 80% methanol (acetonitrile/water = 53:47, flow rate: 0.5 mL/min, column temperature: 15°C); Figure S5. The chromatogram of the ginsenoside fraction obtained from SPE with 80% methanol (acetonitrile/water = 53:47, flow rate: 0.6 mL/min, column temperature: 25 °C); Figure S6. The semi-preparative chromatogram of the ginsenoside fraction obtained from SPE with 80% methanol; Figure S7. ^1^H-NMR spectrum of compound 1 in pyridine-d_5_; Figure S8. ^13^C-NMR spectrum of compound **1** in pyridine-*d*_5;_ Figure S9. HSQC spectrum of compound **1** in pyridine-*d*_5;_ Figure S10. HMBC spectrum of compound **1** in pyridine-*d*_5;_ Figure S11. ROESY spectrum of compound **1** in pyridine-*d*_5;_ Figure S12**.** MS spectra of compound **1**; Figure S13. IR spectrum of compound **1**; Figure S14. ^1^H-NMR spectrum of compound **2** in pyridine-*d*_5_; Figure S15. ^13^C-NMR spectrum of compound **2** in pyridine-*d*_5_; Figure S16. HSQC spectrum of compound **2**; Figure S17. HMBC spectrum of compound **2**; Figure S18. ROESY spectrum of compound **2**; Figure S19. MS spectra of compound **2**; Figure S20. IR spectrum of compound **2**.

## Abbreviations

FBPG	Flower buds of *Panax ginseng*
HMBC	Heteronuclear multiple bond correlation
HSQC	Heteronuclear singular quantum correlation
HR-ESIMS	High resolution electrospray ionization mass spectrometry
LC-MS	Liquid chromatography coupled to mass spectrometry
LOD	Limits of detection
LOQ	Limits of quantification
NMR	Nuclear magnetic resonance
NOE	Nuclear overhauser effect
ROESY	Rotating frame overhauser effect spectroscopy
RSD	Relative standard deviation
RP-HPLC	Reversed-phase high-performance liquid chromatography
S/N	Signal-to-noise
