# Supplementary material for: A Strategy for Simultaneous Isolation of Less Polar Ginsenosides, Including a Pair of New 20-Methoxyl Isomers, from Flower Buds of Panax ginseng
Source: Molecules. 2017 Mar 10;22(3):442. doi: 10.3390/molecules22030442 (PMC6155238; doi:10.3390/molecules22030442)
Supplement: Supplementary file 1 [file molecules-22-00442-s001.pdf]

A strategy for simultaneous isolation of less polar ginsenosides, including a pair of new 20-methoxyl isomers, from flower buds of *Panax ginseng*.

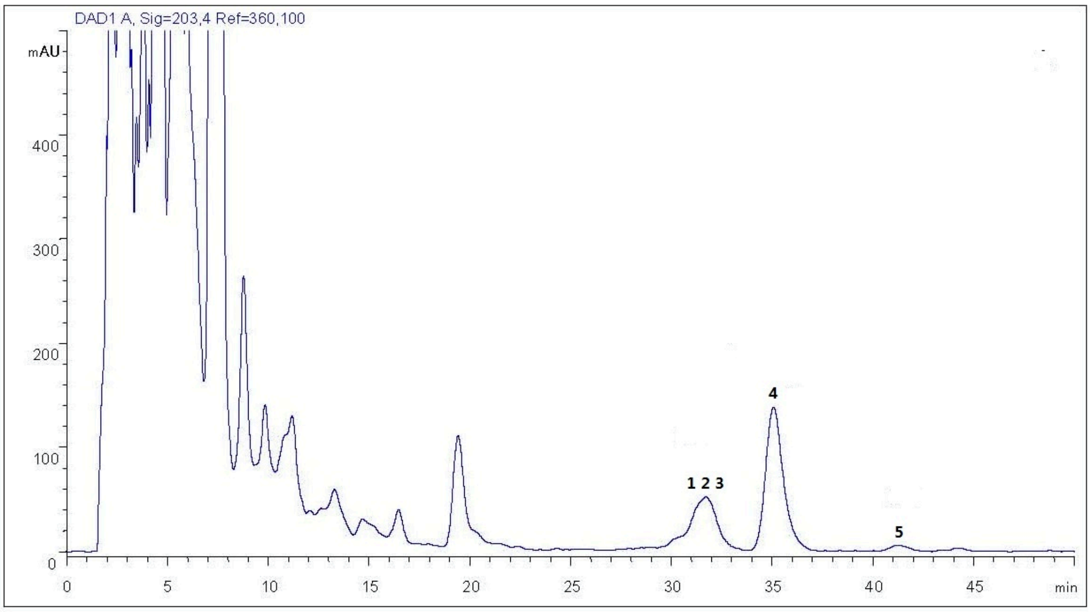

Figure S1. The chromatogram of crude ginsenosides extracts of FBPG after treatment with Diaion HP-20 macroporous resin column (methanol:water = 80:20). Peaks: 1, 20(*S*)-methoxyl-ginsenoside Rg_3; 2, 20(*R*)-methoxyl-ginsenoside Rg_3; 3, ginsenoside Rk_1; 4, ginsenoside Rg_5; 5, ginsenoside M1.

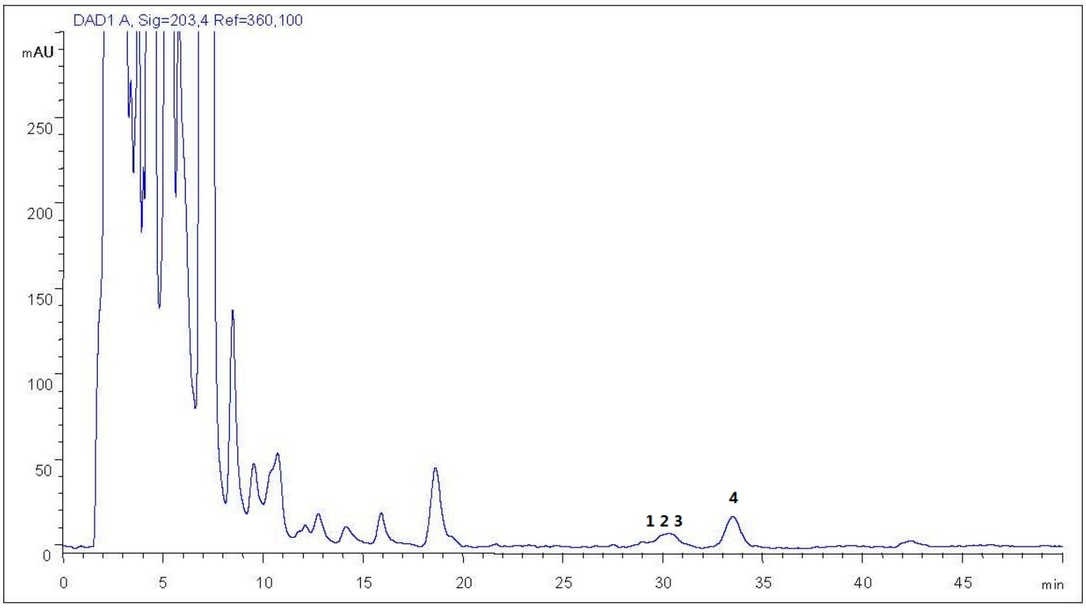

Figure S2. The chromatogram of ginsenosides fraction obtained from SPE with 68% methanol (methanol:water = 80:20).

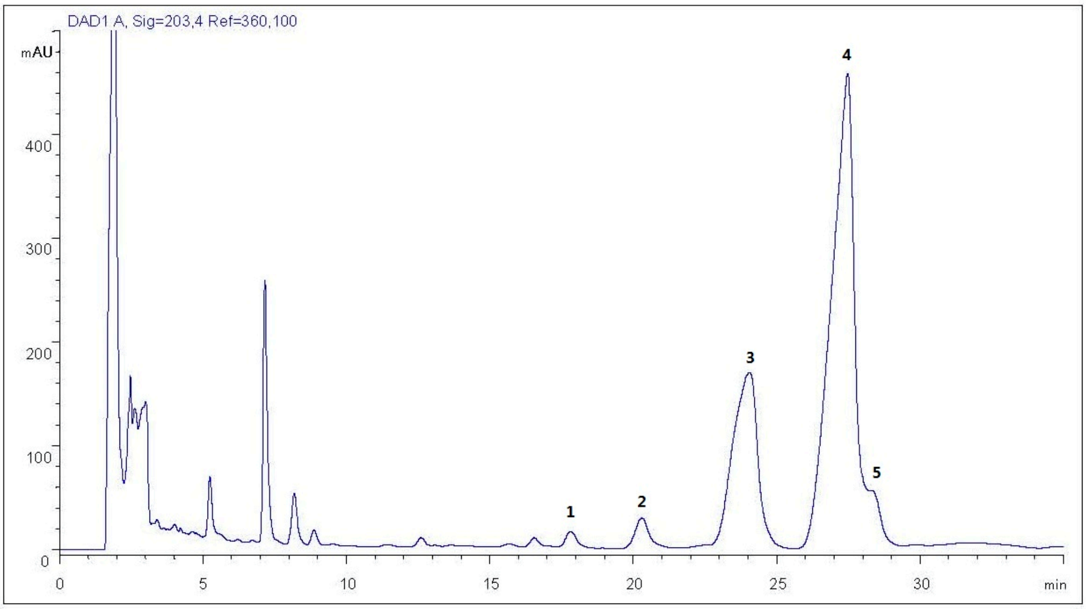

Figure S3. The chromatogram of ginsenosides fraction obtained from SPE with 80% methanol (acetonitrile-water = 50:47, flow rate 0.6 mL/min, column temperature 15°C).

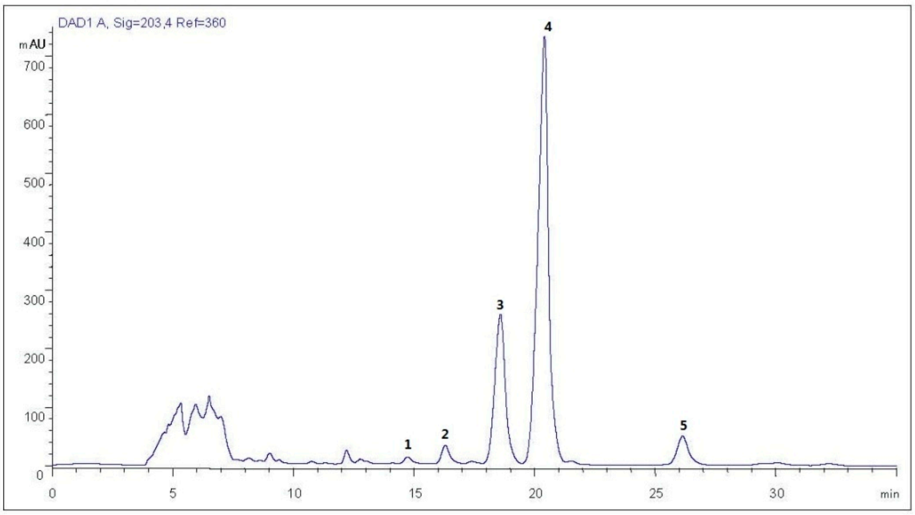

Figure S4. The chromatogram of ginsenosides fraction obtained from SPE with 80% methanol (acetonitrile-water = 53:47, flow rate 0.5 mL/min, column temperature 15°C).

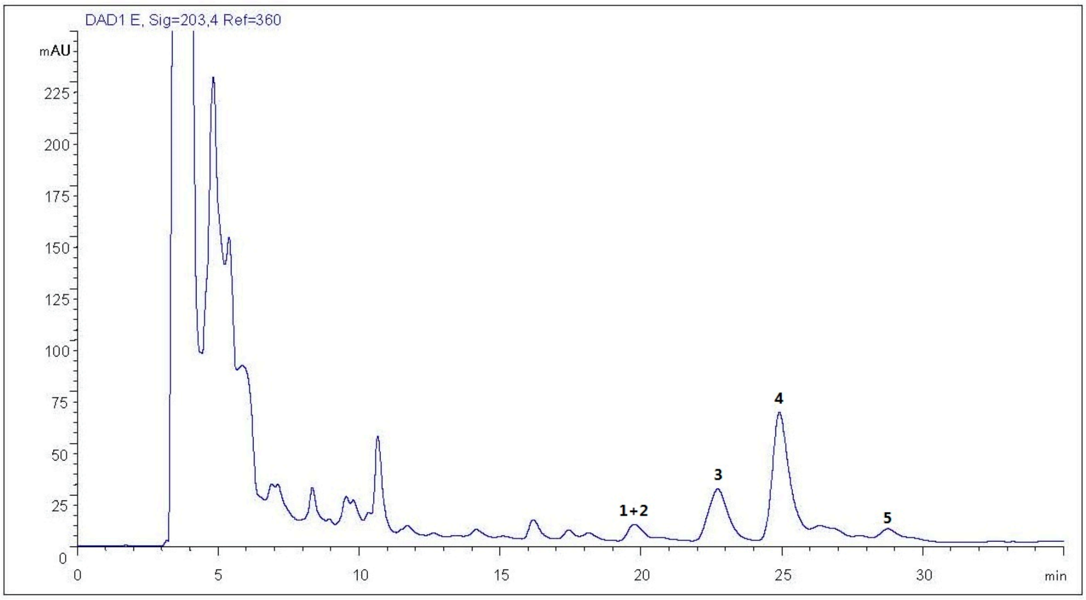

Figure S5. The chromatogram of ginsenosides fraction obtained from SPE with 80% methanol (acetonitrile-water = 53:47, flow rate 0.6 mL/min, column temperature 25°C).

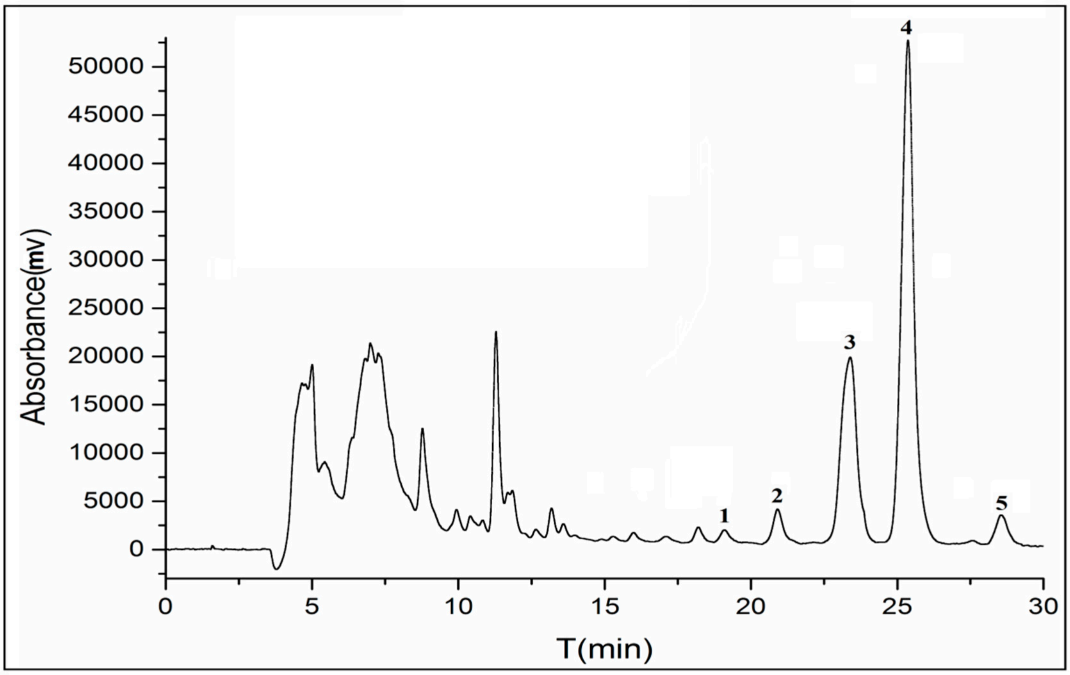

Figure S6. The semi-preparative chromatogram of ginsenosides fraction obtained from SPE with 80% methanol.

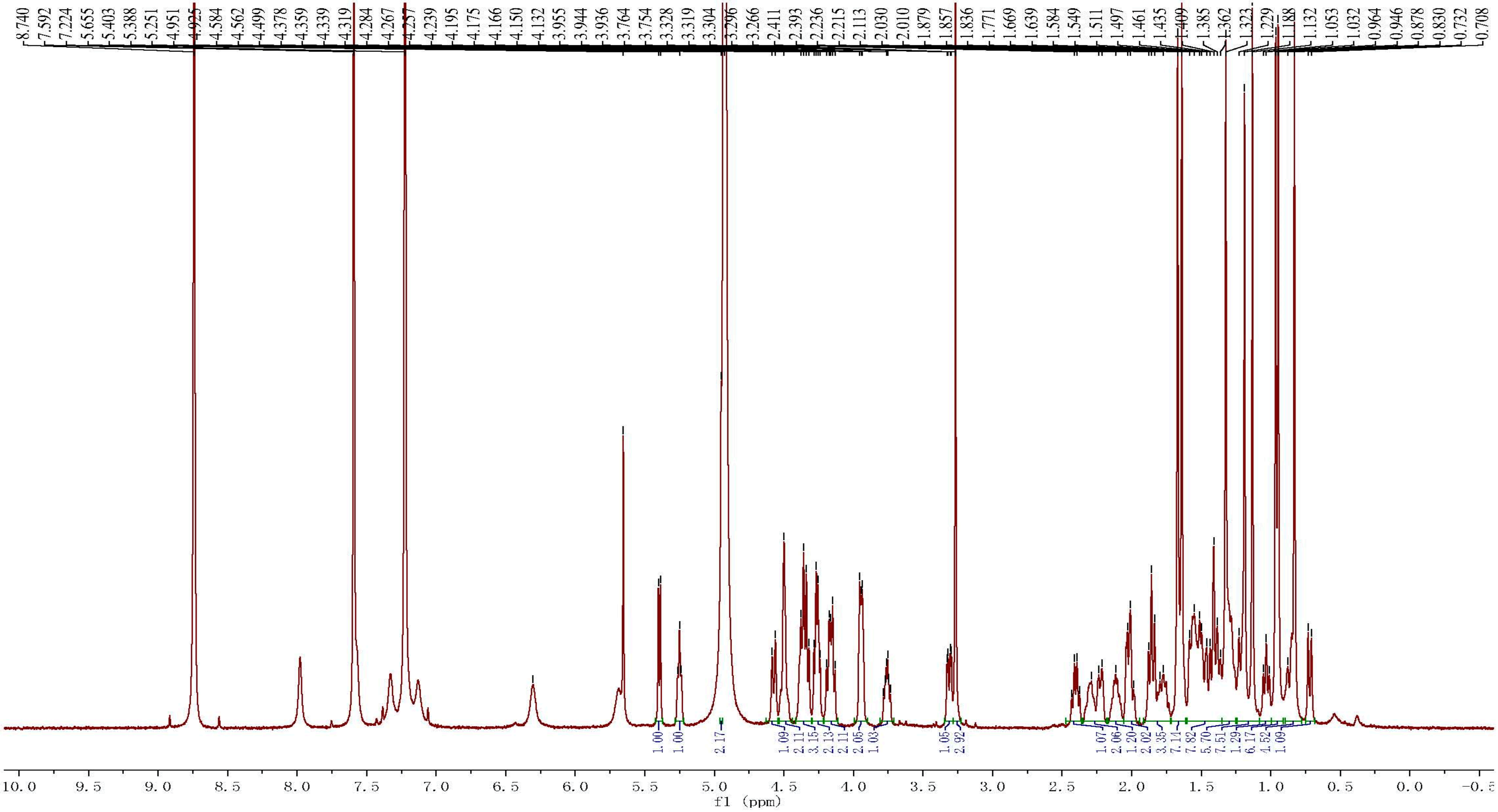

Figure S7. ^{1}H NMR spectrum of compound **1** in pyridine-*d*5.

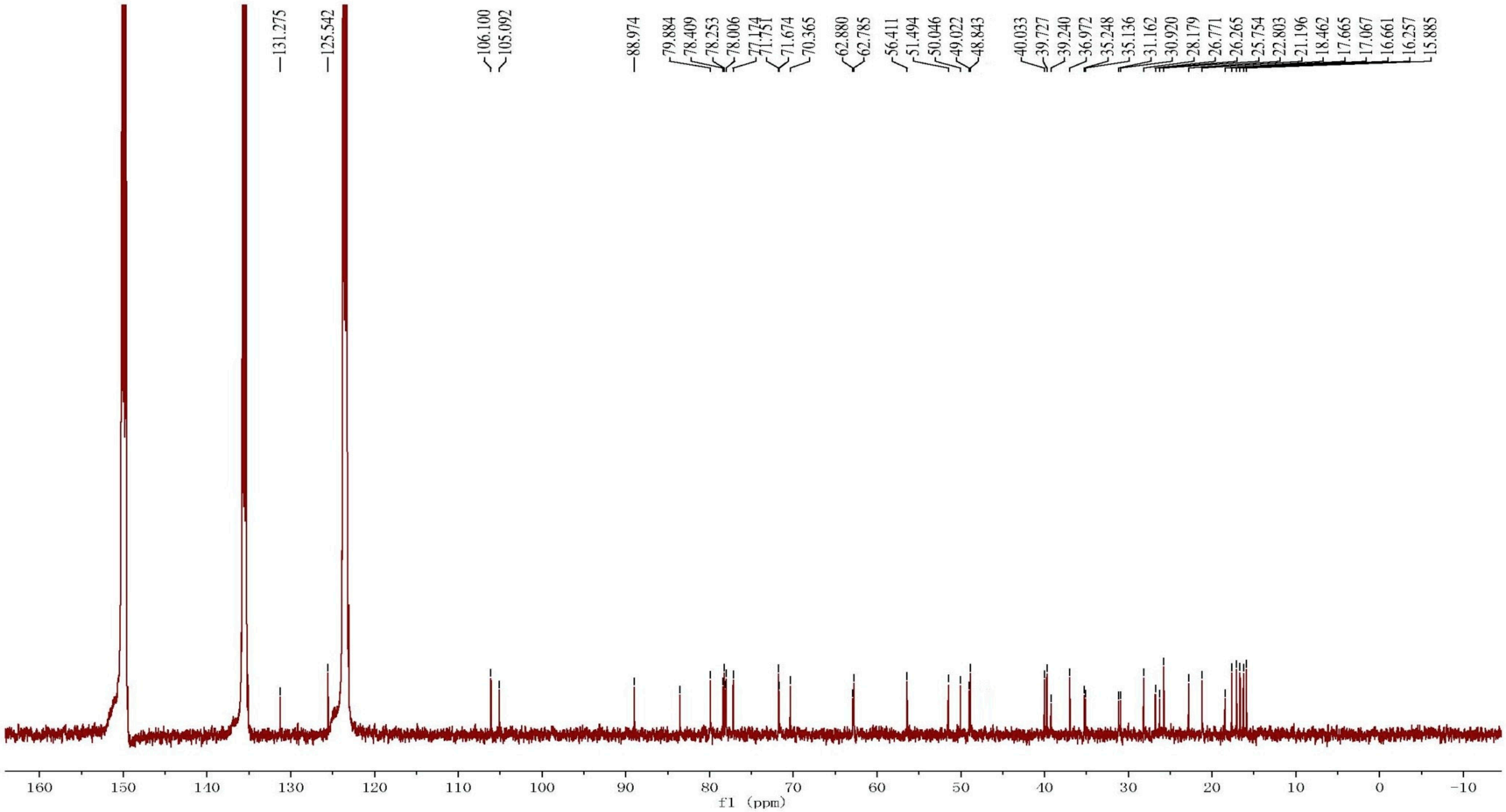

Figure S8. ^{13}C NMR spectrum of compound **1** in pyridine-d_5.

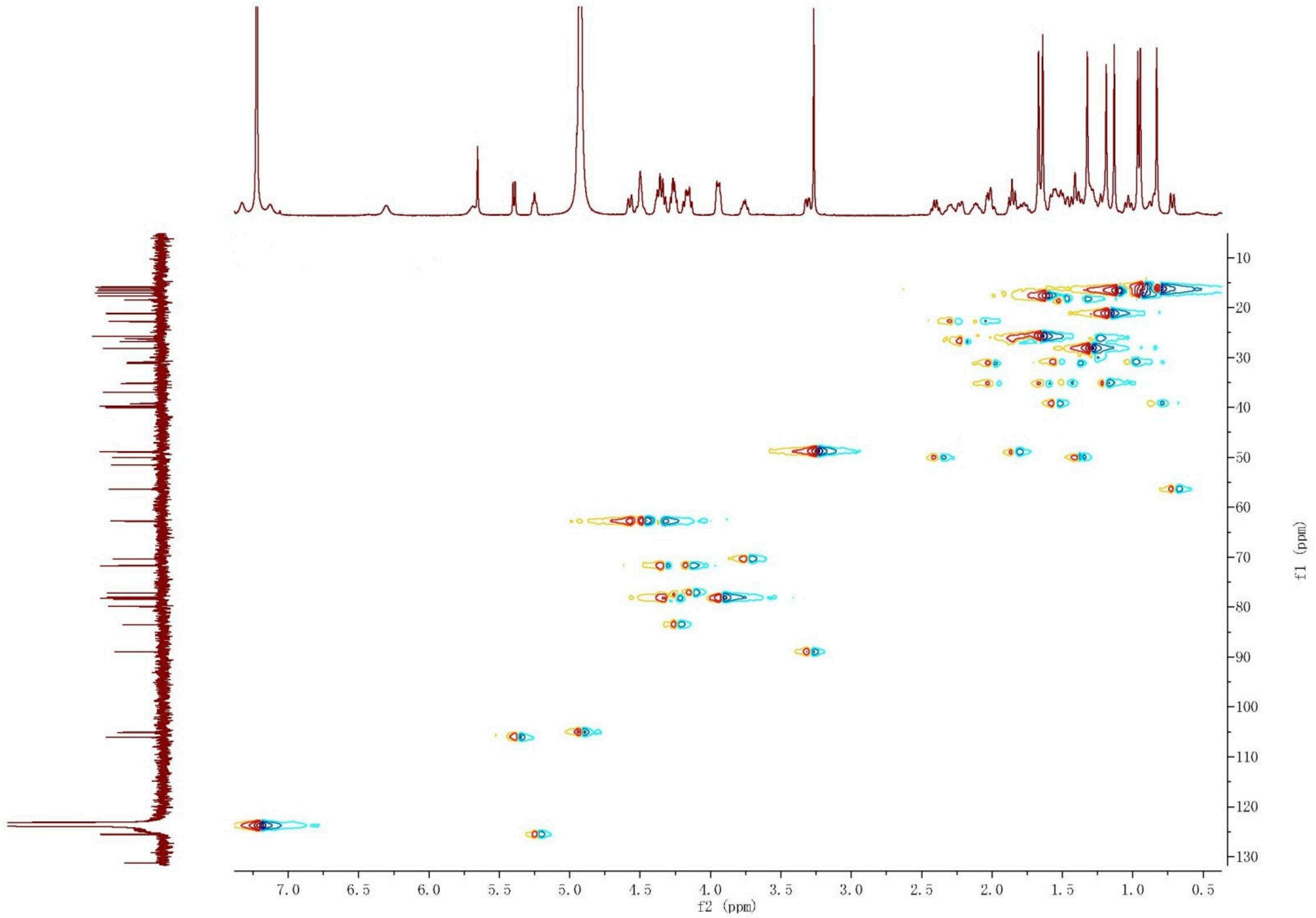

Figure S9. HSQC spectrum of compound **1** in pyridine-d_5.

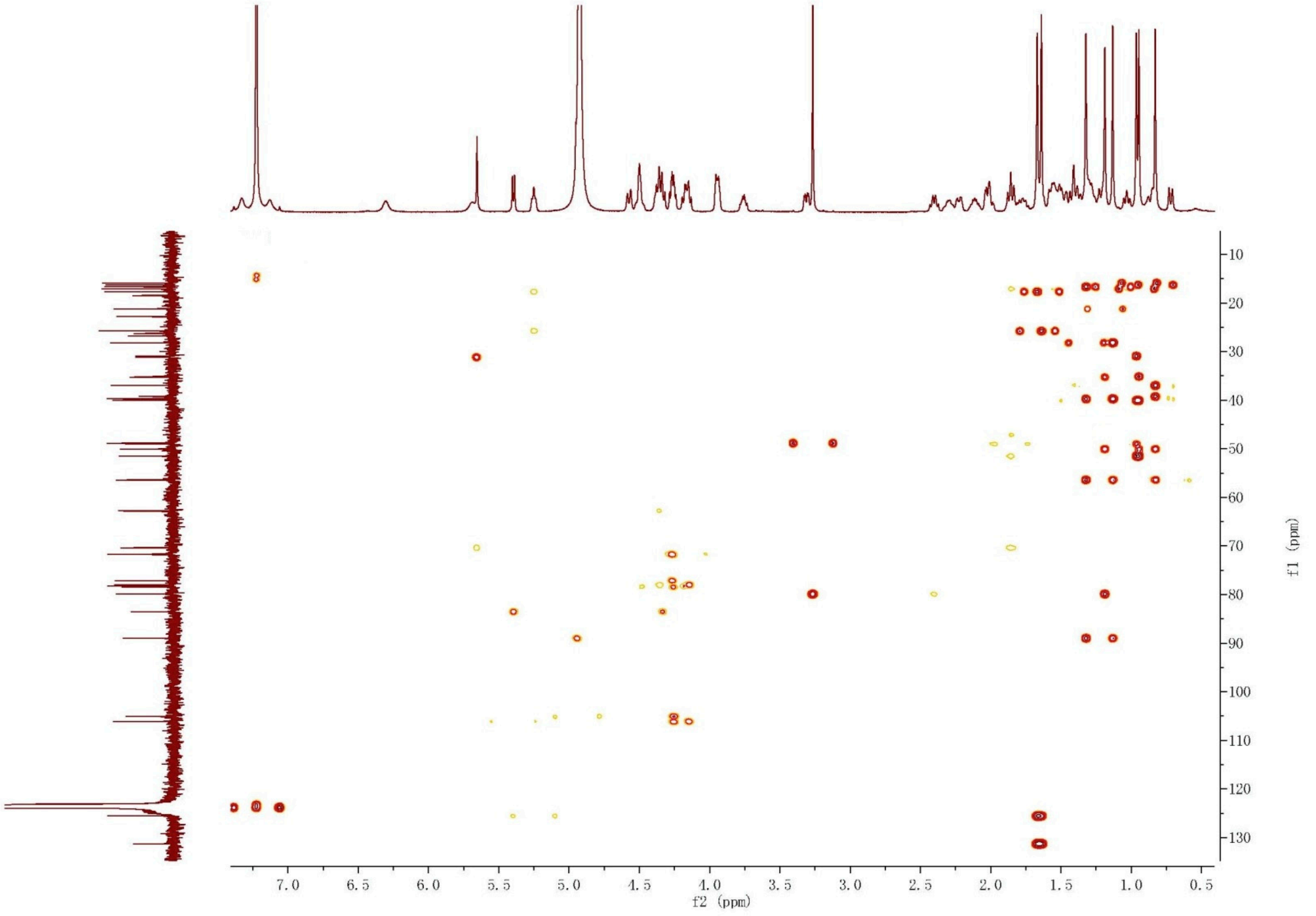

Figure S10. HMBC spectrum of compound **1** in pyridine-d_5.

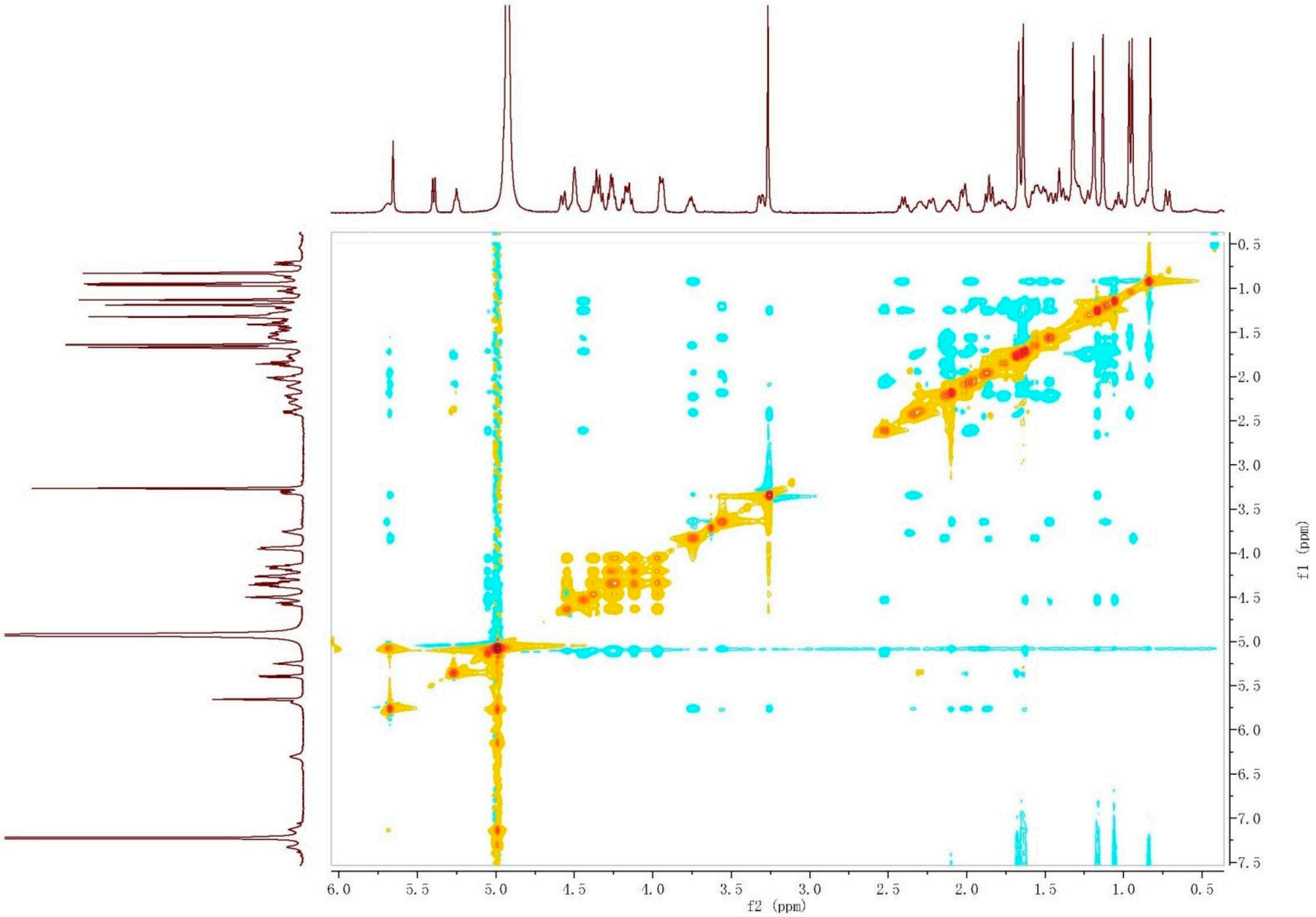

Figure S11. ROESY spectrum of compound **1** in pyridine-d_5.

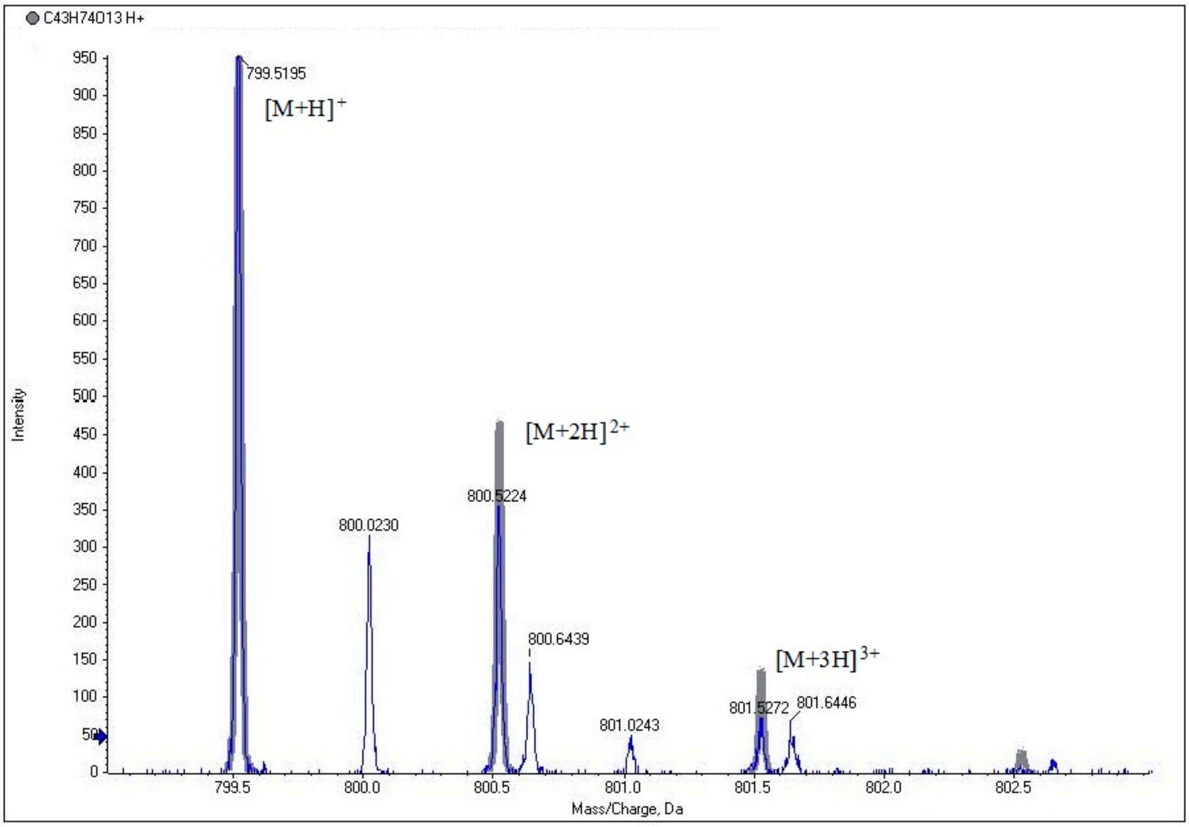

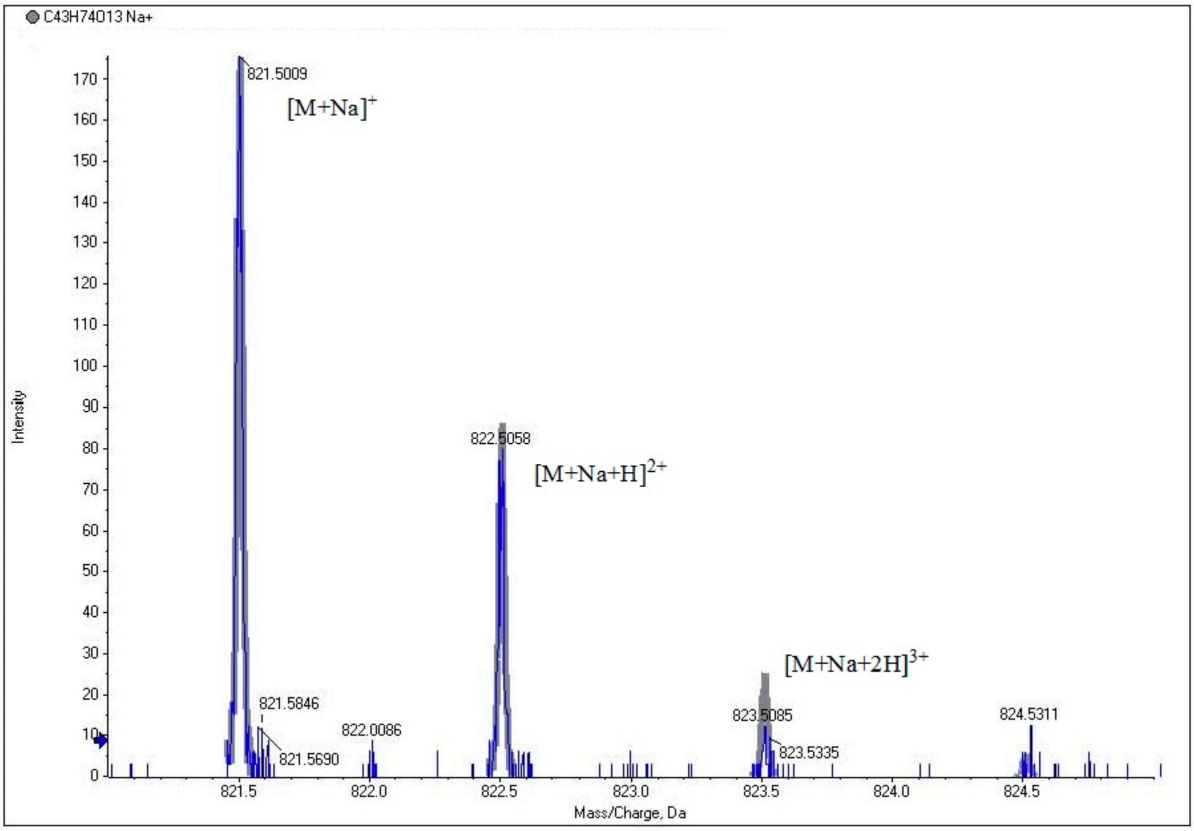

Figure S12. MS spectra of compound **1**.

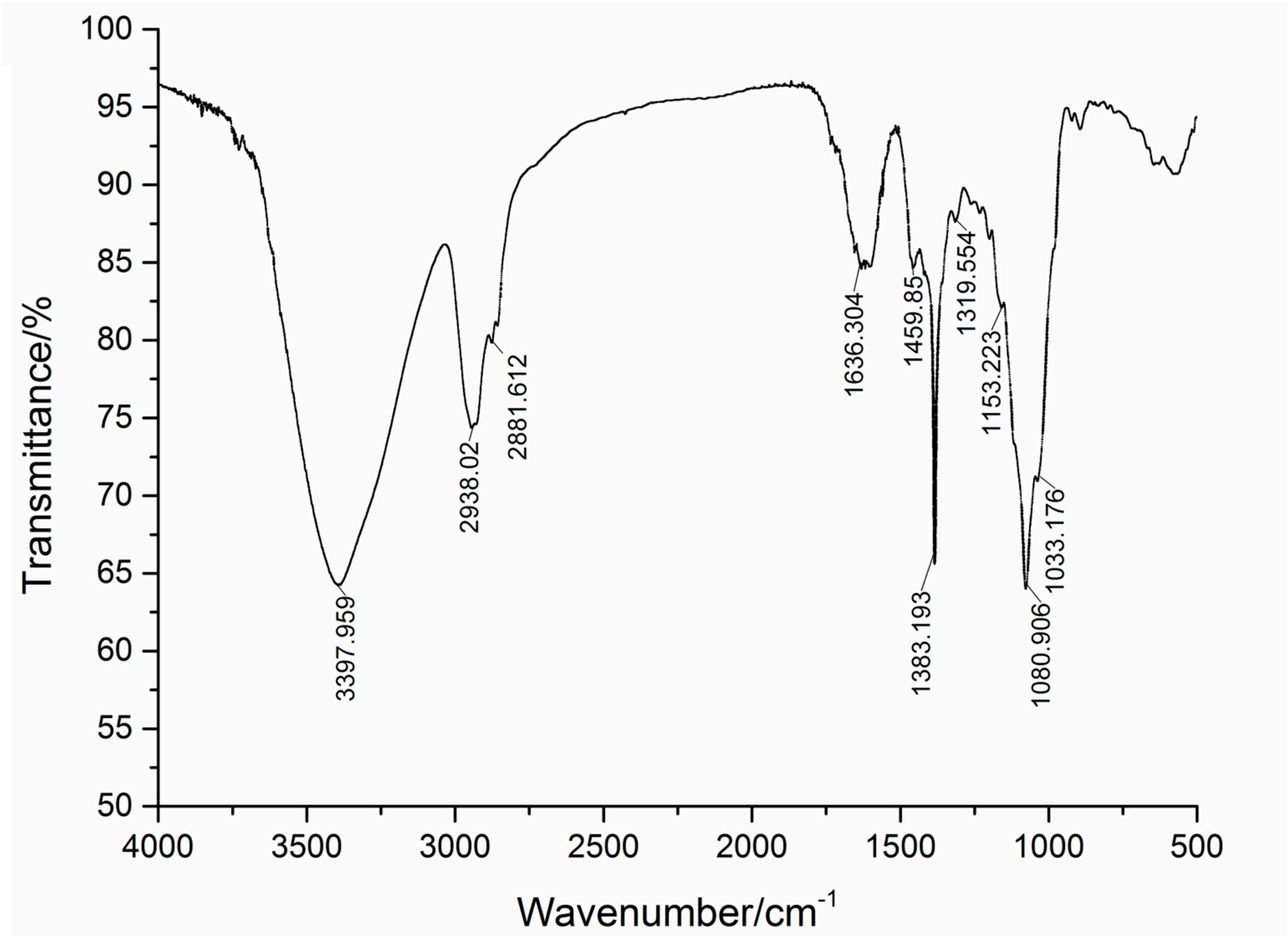

Figure S13. IR spectrum of compound **1**.

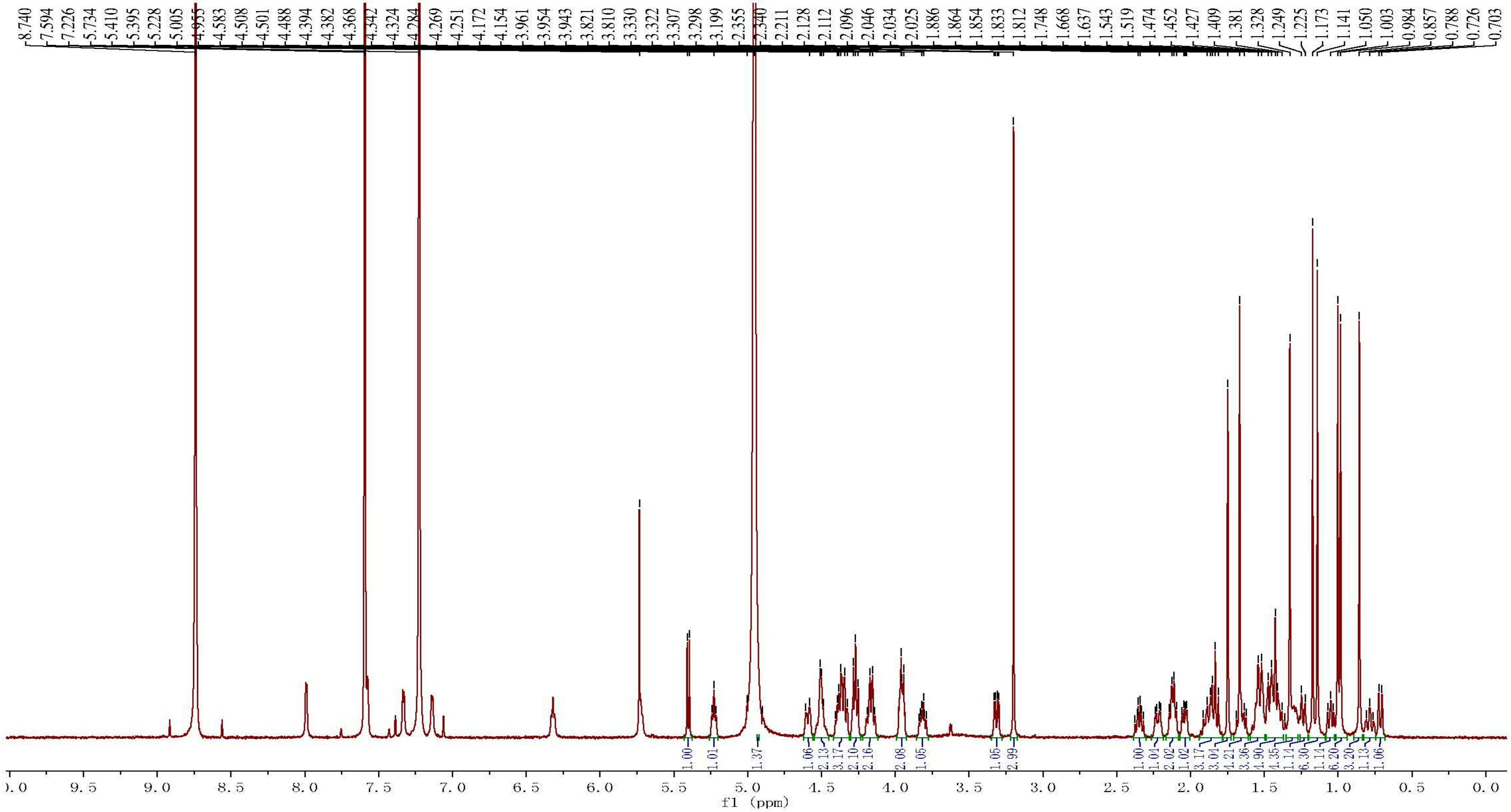

Figure S14. ^{1}H NMR spectrum of compound **2** in pyridine-d_5.

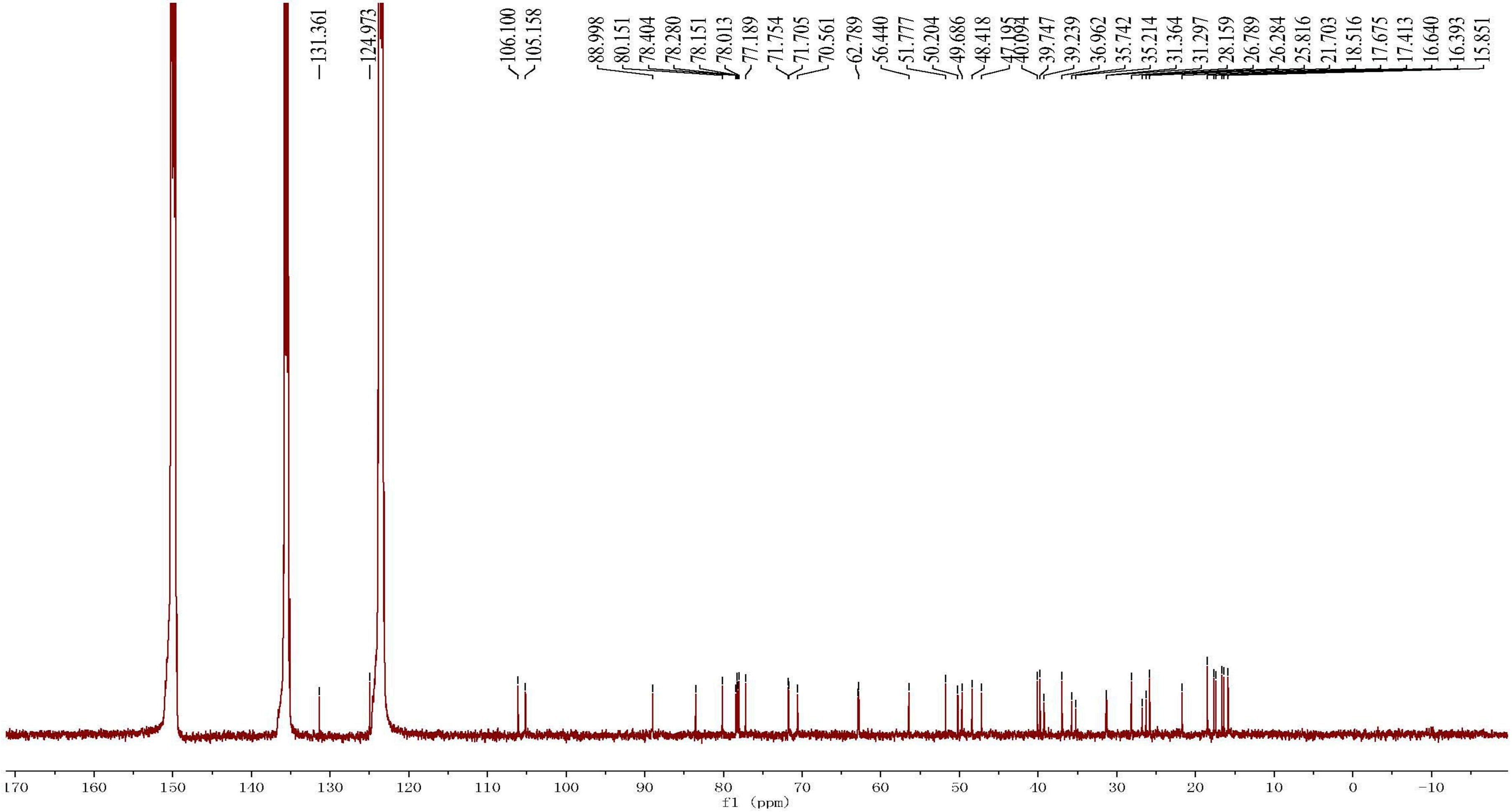

Figure S15. ^{13}C NMR spectrum of compound **2** in pyridine-d_5.

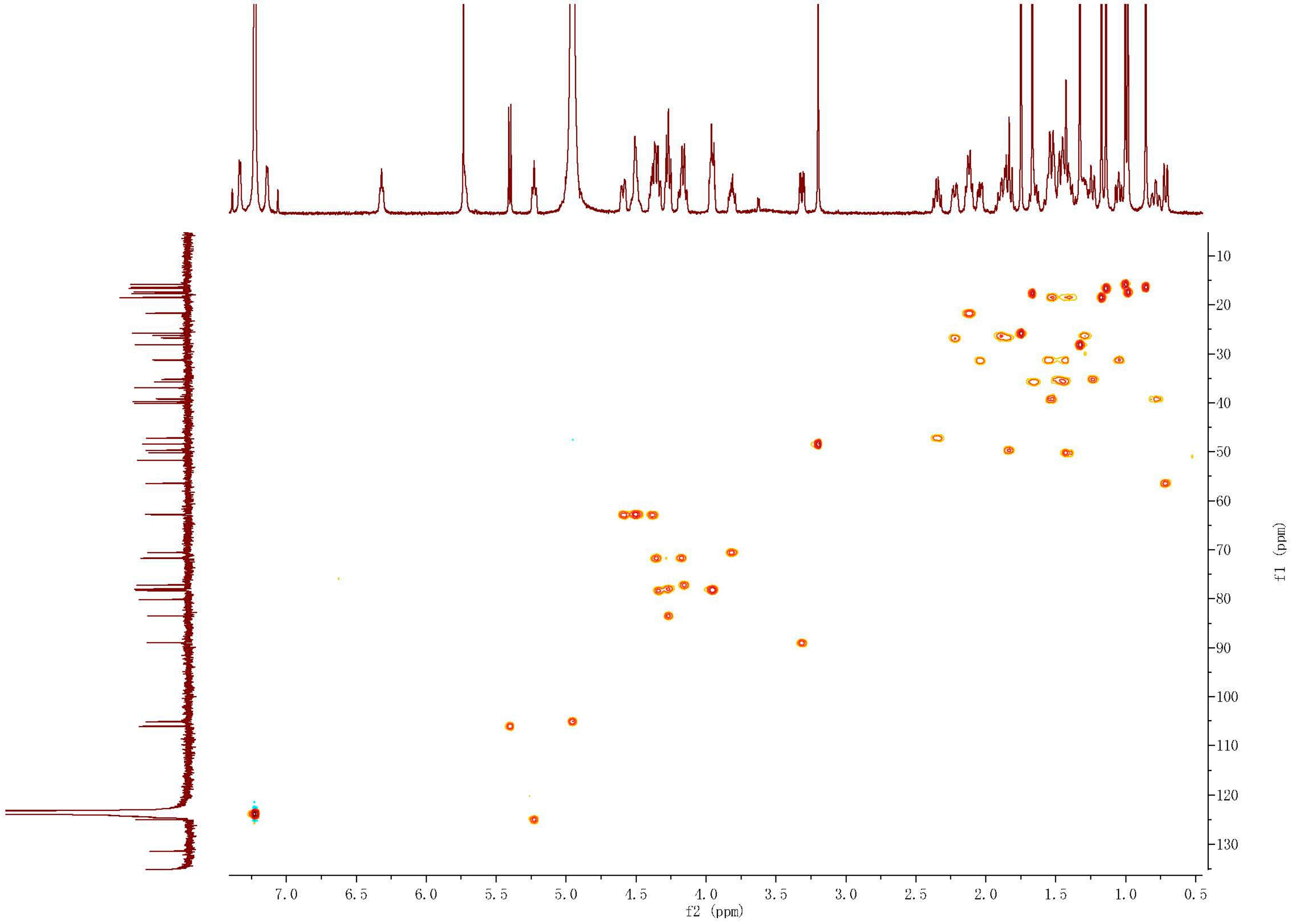

Figure S16. HSQC spectrum of compound **2**.

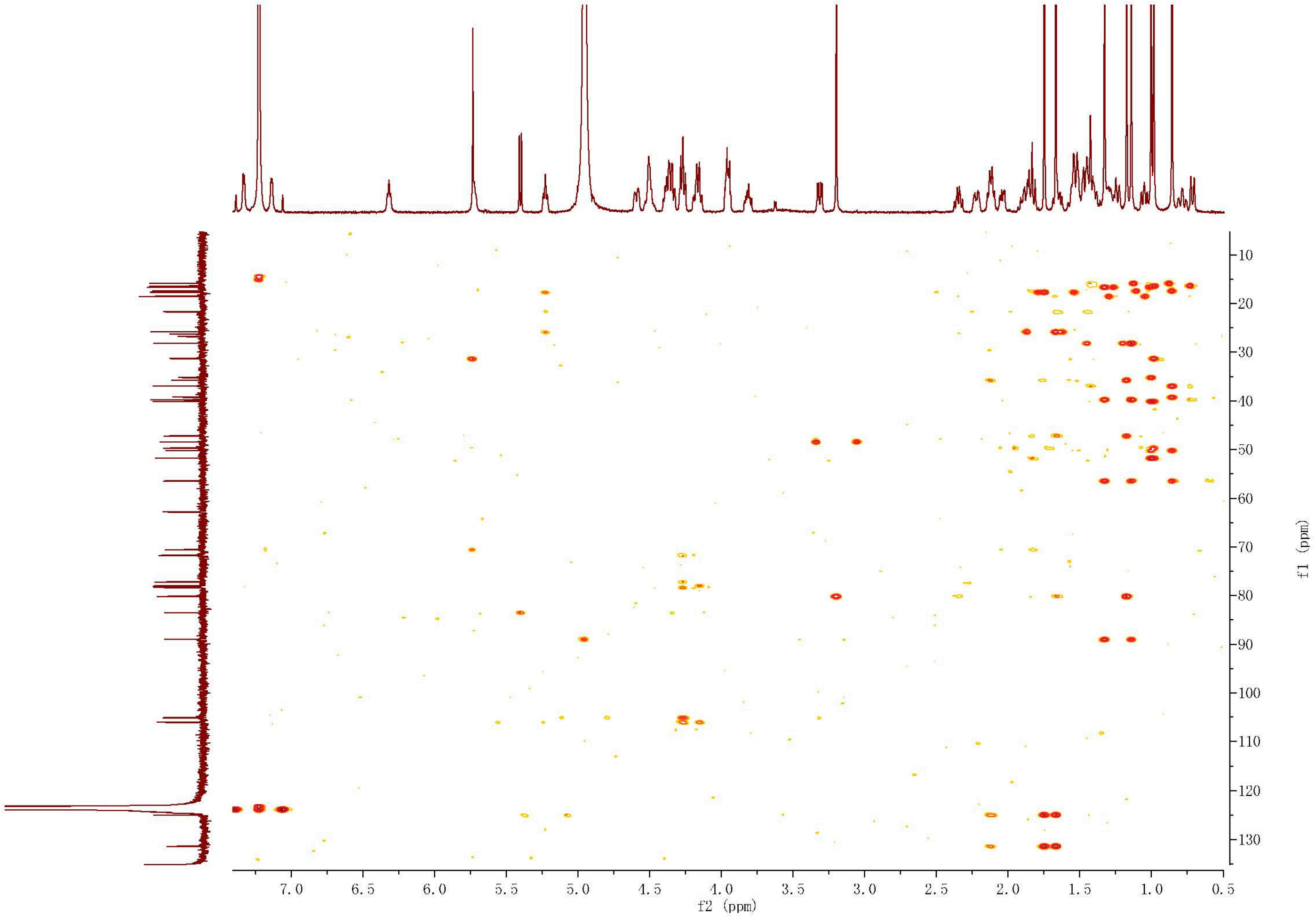

Figure S17. HMBC spectrum of compound **2**

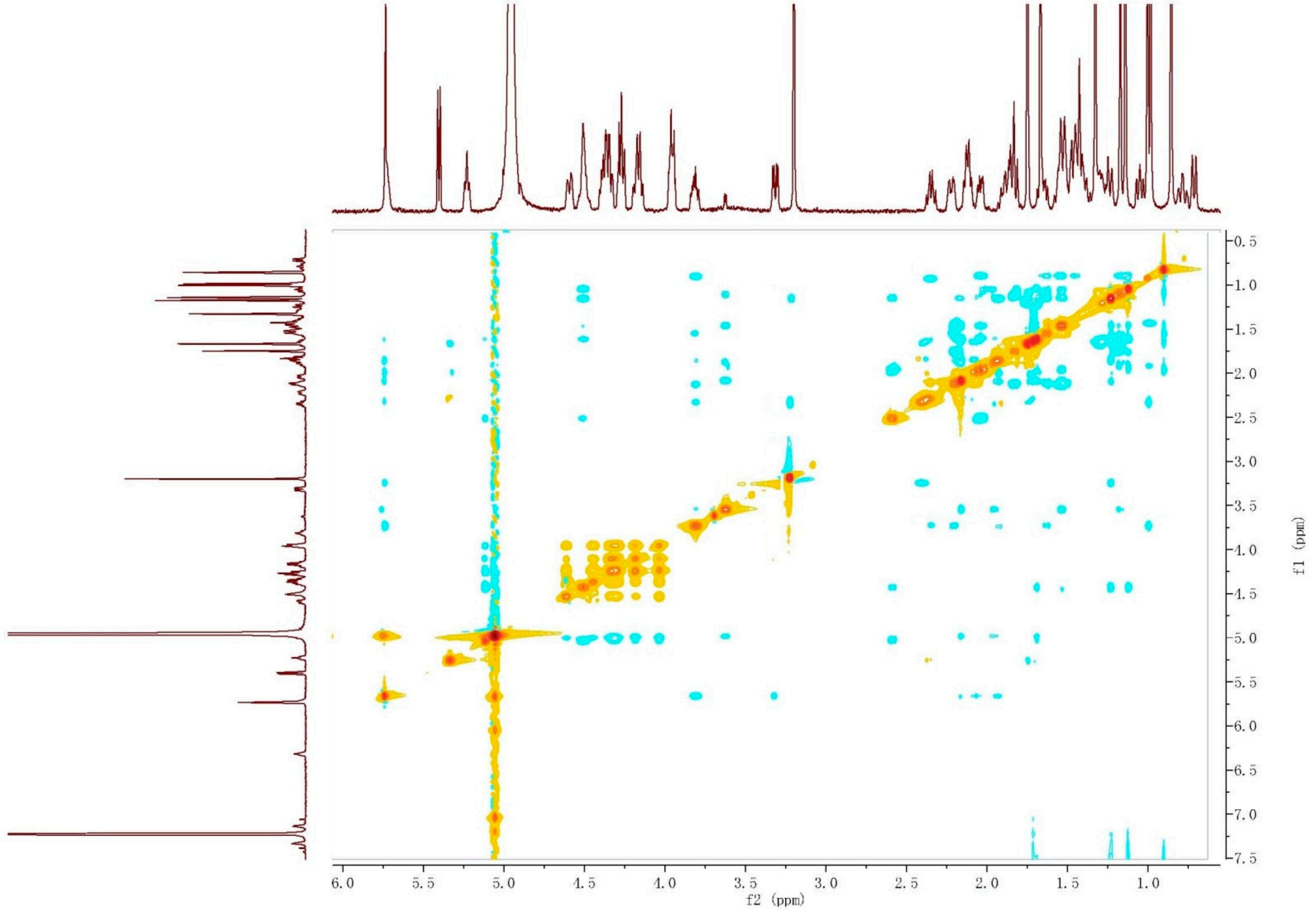

Figure S18. ROESY spectrum of compound **2**.

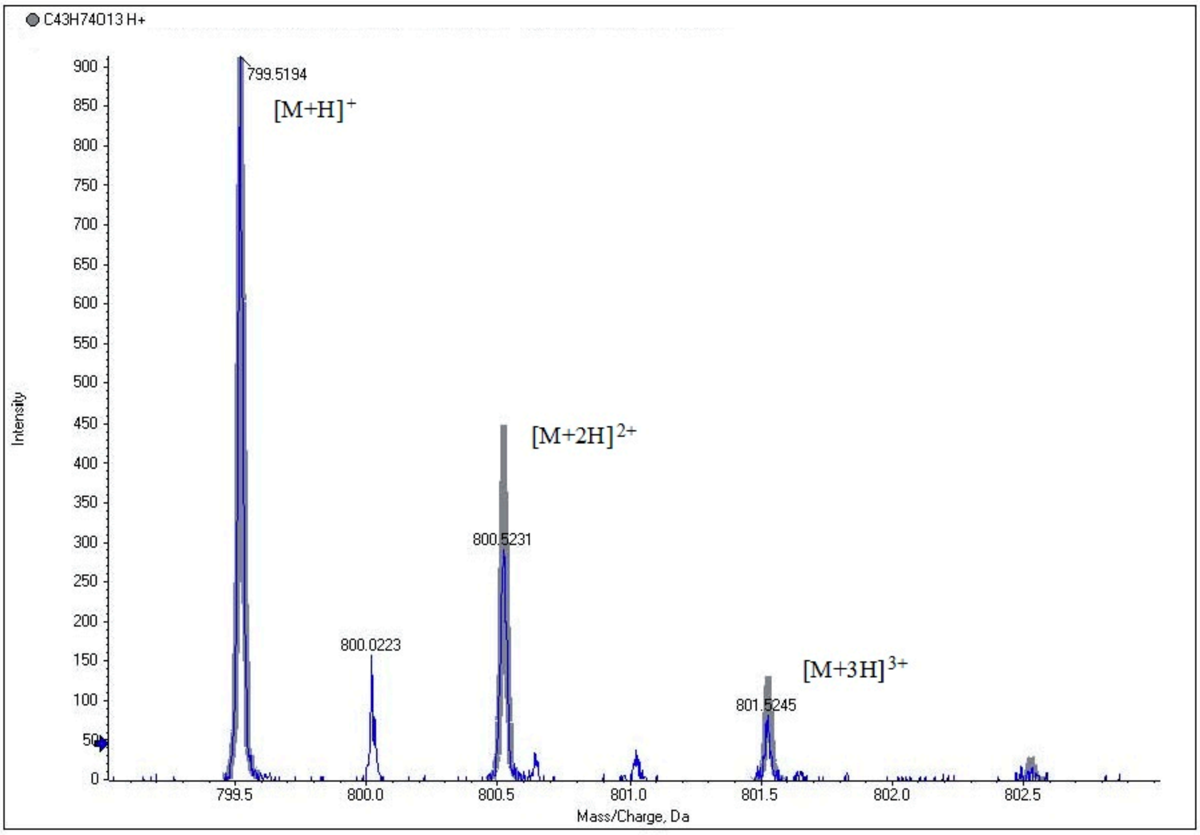

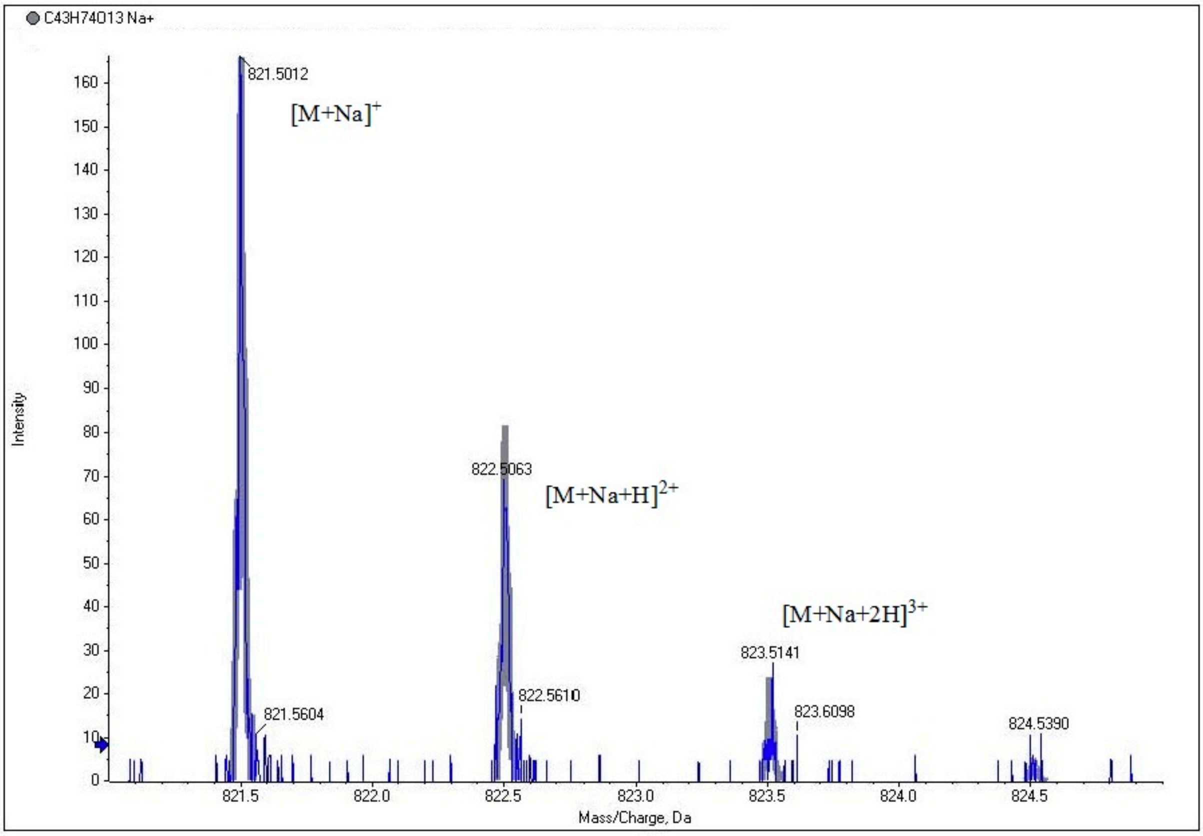

Figure S19. MS spectra of compound **2**.

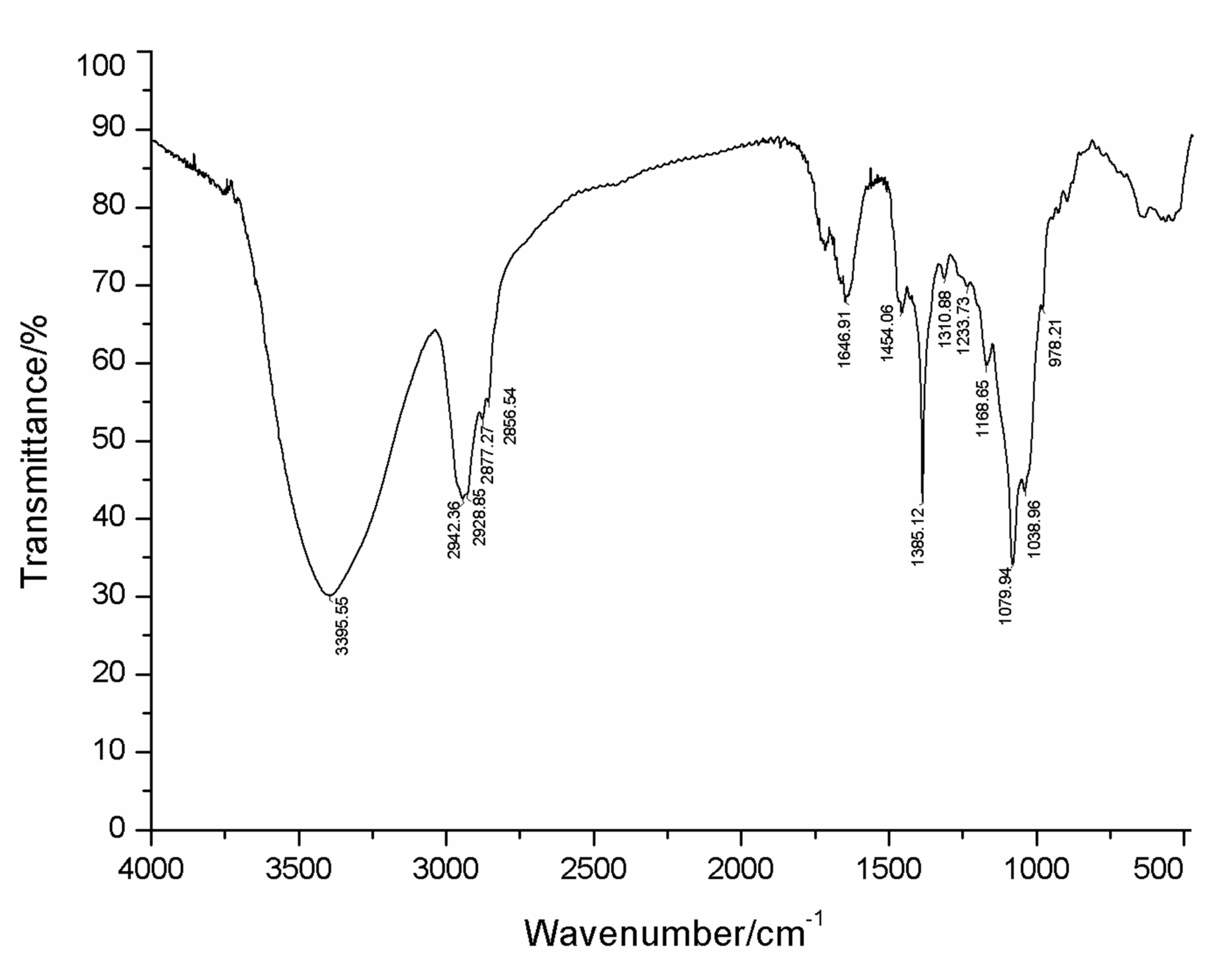

Figure S20. IR spectrum of compound **2.**
